# The diagnostic value of PET/CT imaging with the ^68^Ga-labelled PSMA ligand HBED-CC in the diagnosis of recurrent prostate cancer

**DOI:** 10.1007/s00259-014-2949-6

**Published:** 2014-11-20

**Authors:** Ali Afshar-Oromieh, Eleni Avtzi, Frederik L. Giesel, Tim Holland-Letz, Heinz G. Linhart, Matthias Eder, Michael Eisenhut, Silvan Boxler, Boris A. Hadaschik, Clemens Kratochwil, Wilko Weichert, Klaus Kopka, Jürgen Debus, Uwe Haberkorn

**Affiliations:** 1 Department of Nuclear Medicine, University Hospital of Heidelberg, Im Neuenheimer Feld 400, 69120 Heidelberg, Germany; 2 German Cancer Research Centre, Clinical Cooperation Unit Nuclear Medicine, Heidelberg, Germany; 3 Department of Biostatistics, German Cancer Research Center, Im Neuenheimer Feld 280, Heidelberg, Germany; 4 National Centre for Tumor Diseases (NCT), German Cancer Research Centre, Heidelberg, Im Neuenheimer Feld 581, 69120 Heidelberg, Germany; 5 Division of Radiopharmaceutical Chemistry, German Cancer Research Center, Im Neuenheimer Feld 280, Heidelberg, Germany; 6 Department of Urology, University Hospital of Heidelberg, Im Neuenheimer Feld 110, Heidelberg, Germany; 7 Department of Pathology, University Hospital of Heidelberg, Im Neuenheimer Feld 224, 69120 Heidelberg, Germany; 8 Department of Radiation Oncology and Therapy, University Hospital Heidelberg, Im Neuenheimer Feld 400, 69120 Heidelberg, Germany

**Keywords:** Prostate cancer, PET/CT, Positron emission tomography, PSMA, FMCH, FECH

## Abstract

**Purpose:**

Since the introduction of positron emission tomography (PET) imaging with ^68^Ga-PSMA-HBED-CC (=^68^Ga-DKFZ-PSMA-11), this method has been regarded as a significant step forward in the diagnosis of recurrent prostate cancer (PCa). However, published data exist for small patient cohorts only. The aim of this evaluation was to analyse the diagnostic value of ^68^Ga-PSMA-ligand PET/CT in a large cohort and the influence of several possibly interacting variables.

**Methods:**

We performed a retrospective analysis in 319 patients who underwent ^68^Ga-PSMA-ligand PET/CT from 2011 to 2014. Potential influences of several factors such as prostate-specific antigen (PSA) level and doubling time (DT), Gleason score (GSC), androgen deprivation therapy (ADT), age and amount of injected tracer were evaluated. Histological verification was performed in 42 patients after the ^68^Ga-PSMA-ligand PET/CT. Tracer uptake was measured in 901 representative tumour lesions.

**Results:**

In 82.8 % of the patients at least one lesion indicative of PCa was detected. Tumor-detection was positively associated with PSA level and ADT. GSC and PSA-DT were not associated with tumor-detection. The average maximum standardized uptake value (SUV_max_) of tumour lesions was 13.3 ± 14.6 (0.7–122.5). Amongst lesions investigated by histology, 30 were false-negative in 4 different patients, and all other lesions (*n* = 416) were true-positive or true-negative. A lesion-based analysis of sensitivity, specificity, negative predictive value (NPV) and positive predictive value (PPV) revealed values of 76.6 %, 100 %, 91.4 % and 100 %. A patient-based analysis revealed a sensitivity of 88.1 %. Of 116 patients available for follow-up, 50 received local therapy after ^68^Ga-PSMA-ligand PET/CT.

**Conclusion:**

^68^Ga-PSMA-ligand PET/CT can detect recurrent PCa in a high number of patients. In addition, the radiotracer is highly specific for PCa. Tumour detection is positively associated with PSA and ADT. ^68^Ga-PSMA-ligand PET/CT can help delay systemic therapy of PCa.

**Electronic supplementary material:**

The online version of this article (doi:10.1007/s00259-014-2949-6) contains supplementary material, which is available to authorized users.

## Introduction

Prostate cancer (PCa) is the most frequent tumour entity in men worldwide and an increasing incidence has been noted in recent years [1]. An important problem in clinical management is the development of tumour recurrence after prostatectomy, radiotherapy or other local treatment modalities. In most cases, recurrence after initial therapy is diagnosed either by two consecutive prostate-specific antigen (PSA) values of ≥0.2 μg/l after prostatectomy or external beam radiation therapy [2].

One of the key issues is early detection of recurrent disease. If the tumour is accessible for surgery or external radiation therapy, patients may be cured or systemic therapy and the resultant side effects can be delayed. However, the prerequisite of these approaches is an accurate diagnostic modality with high sensitivity and specificity. To date, this is a major challenge for all conventional imaging methods [3].

Although choline-based positron emission tomography (PET)/CT is widely used for this purpose, there have been numerous studies reporting a low sensitivity and specificity, especially at low PSA levels and high Gleason scores (GSC) [4–8]. Therefore, the development of new and improved imaging methods was required. In this context, the prostate-specific membrane antigen (PSMA) has recently received increased attention [9–13]. This cell surface protein is significantly overexpressed in PCa cells compared to other PSMA-expressing tissues such as kidney, proximal small intestine or salivary glands [14, 15]. It therefore provides a promising target for PCa-specific imaging and therapy [10, 11, 16–23].

Amongst the methods available to image PSMA-expressing tumours, Glu-NH-CO-NH-Lys-(Ahx)-[^68^Ga(HBED-CC)] (^68^Ga-DKFZ-PSMA-11) as a ^68^Ga-labelled PSMA-targeted radioligand developed by our group became one of the most successful with respect to clinical application showing a rapid spread across many countries. However, to date data exist only for a limited number of patients, and the influence of several parameters used for follow-up and assessment of prognosis has not been addressed. The aim of this evaluation was to retrospectively analyse a greater number of patients investigated with this imaging modality as well as possibly interacting variables such as PSA level, GSC, androgen deprivation therapy (ADT), age and amount of injected radiotracer.

## Materials and methods

For this evaluation we performed a retrospective analysis of 319 patients (Table 1 and Supplementary Table 1) who underwent PET/CT 1 h post-injection (p.i.) of ^68^Ga-PSMA-HBED-CC (=^68^Ga-DKFZ-PSMA-11) between May 2011 and January 2014 at our department. All patients signed a written informed consent form for the purpose of anonymized evaluation and publication of their data. All reported investigations were conducted in accordance with the Helsinki Declaration and with our national regulations. This evaluation was approved by the Ethics Committee of the University of Heidelberg (permit S-321/2012). One hundred and two patients in the present evaluation had been partially analysed in previous studies [18–21, 24–26]. The authors are convinced that these patients had to be included in the present evaluation instead of being excluded. Their inclusion improves the data quality and significantly strengthens statistical analysis and conclusions. Patients who did not sign the written informed consent (*n* = 19) and those who were investigated outside a time frame of 45–75 min p.i. (*n* = 18) were excluded from this evaluation.

**Table 1:** Characteristics of the patients investigated in this study (for more details, see Supplementary Table 1)

	Age (y) [*n* = 319]	Tracer (MBq) [*n* = 319]	GSC [*n* = 284]	PSA at PET [*n* = 311]
Mean	67.6	168	7.5	161.0
SD	7.1	71.4	1.1	2347.6
Range	46-86	40–400	5–10	0.01–41395
Median	68	154	7	4.59
Prostatectomy		Radiation Therapy^a^		ADT at PET
Yes = 226		Yes = 177		Yes = 86
No = 89		No = 125		No = 233
n/a = 4		n/a = 17		n/a = 0
PSA Doubling-Time [n/a: *n* = 127]				
0–1 month (*n* = 30)	1–3 months (*n* = 52)	3–6 months (*n* = 36)	6–12 months (*n* = 34)	>1 year (*n* = 37)
Initial Pathological Stage (TNM) [Incomplete TNM: *n* = 44. n/a: *n* = 91]				
pT1 pN0 (*n* = 4)	pT2 pN0 (*n* = 61)	pT3a pN0 (*n* = 30)	pT3b/pT4 + pN0 (*n* = 29)	any pT and pN+ (*n* = 60)

*SD* standard deviation

^a^Radiation therapy of the prostate gland or of the prostate fossa after prostatectomy

In most patients (*n* = 292) progressive disease was suspected following prior conventional treatment of PCa (e.g. radiation therapy and/or surgery). In 27 cases, PET/CT was conducted before initiation of local therapy to exclude metastases after PCa was confirmed by biopsy and in 38 cases (also progressive disease) to evaluate possible therapy with radiolabelled PSMA ligands [21]. Patients with very low PSA values were referred to PSMA imaging after progressive disease was suspected in alternative imaging modalities such as computed tomography (CT) or magnetic resonance imaging (MRI).

To increase the validity of the statistical analysis, only the initial PET/CT of each patient was included in this evaluation, regardless of whether they were referred for more scans during follow-up. The analysed patients were therefore not strictly consecutive.

Amongst all lesions visually considered typical for PCa (for more details, see the “Imaging” section), we selected 901 representative lesions for radiotracer uptake analysis measured in maximum standardized uptake value (SUV_max_) as this is common in routine clinical practice. Any visible PCa lesion of a patient was counted and analysed unless they had more than ten lesions. In such a case a maximum of ten lesions were analysed after random selection. This kind of selection avoids an overestimation of SUV values as otherwise dominant lesions would be preferentially selected. In addition, since ^68^Ga-PSMA-ligand PET/CT was introduced, we learned that any uptake of ^68^Ga-PSMA-HBED-CC in lesions above local background is highly specific for PCa and must therefore be regarded as PCa unless otherwise proven (see also histological results). Therefore, a quantitative cut-off for PCa lesions does not exist.

Forty-two patients with pathological radiotracer uptake in ^68^Ga-PSMA-ligand PET/CT were further investigated by biopsy or surgery at our hospital. Biopsies and operative findings not conducted at our hospital were not included in this evaluation due to insufficient access to the pathological reports. The histology results were compared with the findings of the ^68^Ga-PSMA-ligand PET/CT. An experienced physician from the Department of Urology was involved in this comparison. A lesion-based analysis of sensitivity, specificity, negative predictive value (NPV) and positive predictive value (PPV) was performed. With regard to a patient-based analysis, calculating specificity, NPV and PPV is not possible as we assume that virtually all patients referred to our hospital had recurrent disease or persistent primary tumour and that our cohort therefore did not include true-negative cases. Under this assumption, a patient-based sensitivity of the ^68^Ga-PSMA-ligand PET/CT was calculated, however, with limited validity as no standardization existed for operative approaches and histology. Thus, no true gold standard for patient-wise positivity/negativity existed. In particular, a negative local histology did not mean that no tumour existed in patients. Follow-up could be conducted in 116 patients up to the time this manuscript was submitted.

We also investigated whether the uptake of the radiotracer in salivary glands can be reduced by a sour excretion stimulus. For this reason, ten patients orally received 10 ml of a vitamin C solution every 30 min (overall three times) after the first scan (1 h p.i.) and were then investigated by a second scan 3 h p.i. The uptakes (SUV_mean_ and SUV_max_) of the salivary glands were then measured at 1 and 3 h p.i. and compared to our previous evaluation [19]. The vitamin C solution was prepared by adding 3 g of vitamin C powder to 10 ml of water. In addition, the patients were asked to drink 1 l of water between both scans.

### Imaging

^68^Ga^3+^ was obtained from a ^68^Ge/^68^Ga radionuclide generator and used for radiolabelling of PSMA-HBED-CC as previously described [10, 11]. The final product was formulated in isotonic phosphate-buffered saline (PBS) and sterile filtered. The radiolabelling and purification of the PSMA ligand was performed with an automated radiosynthesizer. Typically, the radiochemical yield is >98 % as determined by radiosynthesizer validation. The ^68^Ga-PSMA-HBED-CC solution was applied to the respective patient via an intravenous bolus injection (mean of 172.4 MBq ±70.9, range 40–400 MBq, median 161 MBq). The targeted activity of ^68^Ga-PSMA-HBED-CC was 2 MBq/kg body weight. Variation of injected radiotracer activity was caused by the short physical half-life of ^68^Ga (68 min) and variable elution efficiencies resulting during the lifetime of the ^68^Ge/^68^Ga generator. However, in our experience with ^68^Ga-PSMA-ligand PET/CT during the last 3 years, all injected activities were sufficient in detecting PCa. All injections contained 2 nmol PSMA ligand resulting in a median specific radioactivity of 80.5 GBq/μmol.

A non-contrast-enhanced CT scan was performed 1 h post tracer injection using the following parameters: slice thickness of 5 mm, increment of 1.5 mm, soft tissue reconstruction kernel, 130 keV and 80 mAs. Immediately after CT scanning, a whole-body PET was acquired in 3-D (matrix 168×168). For each bed position (16.2 cm, overlapping scale 4.2 cm) a 4-min acquisition time with a 15.5-cm field of view (FOV) was used. The emission data were corrected for randoms, scatter and decay. Reconstruction was conducted with an ordered subset expectation maximization (OSEM) algorithm with 2 iterations/8 subsets and Gauss-filtered to a transaxial resolution of 5 mm at full-width at half-maximum (FWHM). Attenuation correction was performed using the low-dose non-enhanced CT data. PET and CT were performed using the same protocol for every patient on a Biograph 6 PET/CT scanner (Siemens, Erlangen, Germany). Image analysis was performed using an appropriate workstation and software (Syngo TrueD, Siemens, Erlangen, Germany).

Two board certified specialists in nuclear medicine with 10 and 9 years of clinical experience (first and third author) read all data sets independently and resolved any disagreements by consensus. Lesions that were visually considered as suggestive for PCa were counted and analysed with respect to their localization (local relapses, lymph node, bone and soft tissue metastases) and their SUV_max_ as it is common practice in the daily routine. SUV_max_ was chosen due to its higher reproducibility between different investigators when compared to SUV_mean_. The latter is always dependent on the volume of interest (VOI) drawn by the investigator, whereas SUV_max_ behaves independently [19]. However, with regard to the evaluation of tracer uptake in salivary glands prior to and after the excretion stimulus, SUV_mean_ was also measured to ensure a detailed comparison to the results of our previous evaluation [19]. For calculation of the SUV, circular regions of interest were drawn around areas with focally increased uptake in transaxial slices and automatically adapted to a 3-D VOI at a 70 % isocontour.

### Statistical analysis

For statistical analysis, Excel 2010 (Microsoft, Redmond, WA, USA), SigmaPlot version 12 software (Systat Software Inc., Chicago, IL, USA) and R version 3.01 were used. In all cases a *p* value of <0.05 was considered statistically significant. The following statistical analyses were used:

1. The association between positive (pathological) PET/CT results and the variables hormonal therapy (at the time of PET/CT), age, amount of injected tracer, PSA level and GSC was investigated, both univariately (*n* = 319 for hormonal therapy, *n* = 319 for age and injected tracer, *n* = 311 for PSA and *n* = 284 for GSC) and multivariately (*n* = 277) using logistic regression.
  GSC was included in this analysis in four different classes: GSC 5–6 (as reference, all other GSC classes were compared to GSC 5–6), GSC 7, GSC 8 and GSC 9–10.
  Injected tracer amount was included as multiples of 100 MBq. The odds ratios therefore refer to changes of 100 MBq.
  Similar to injected tracer amount, age was included as multiples of 10 years.
  As PSA levels showed a highly skewed distribution, they were converted to a natural logarithmic scale (log PSA).
2. The association between positive (pathological) PET/CT results and PSA doubling time (DT) was also investigated, both univariately (*n* = 189) and multivariately (*n* = 171) using logistic regression. As the inclusion of the PSA DT to the multivariate analysis of all other variables would reduce the patient cohort significantly (from 277 to 171), we decided to analyse this variable separately (Supplementary Table 2). PSA DT was included in this analysis in five different classes: up to 1 month (*n* = 30, as reference, all other PSA DT classes were compared to PSA DT up to 1 month), 1–3 months (*n* = 52), 3–6 months (*n* = 36), 6–12 months (*n* = 34) and >1 year (*n* = 37).
3. Proportions of positive PET results were calculated for subgroups defined by PSA levels and GSC. Exact binomial 95 % confidence intervals were determined for these estimates.
4. A two-sided unpaired two-sample *t* test was used to evaluate differences concerning GSC between groups with and without pathological uptakes.

## Results

**Fig. 1:**
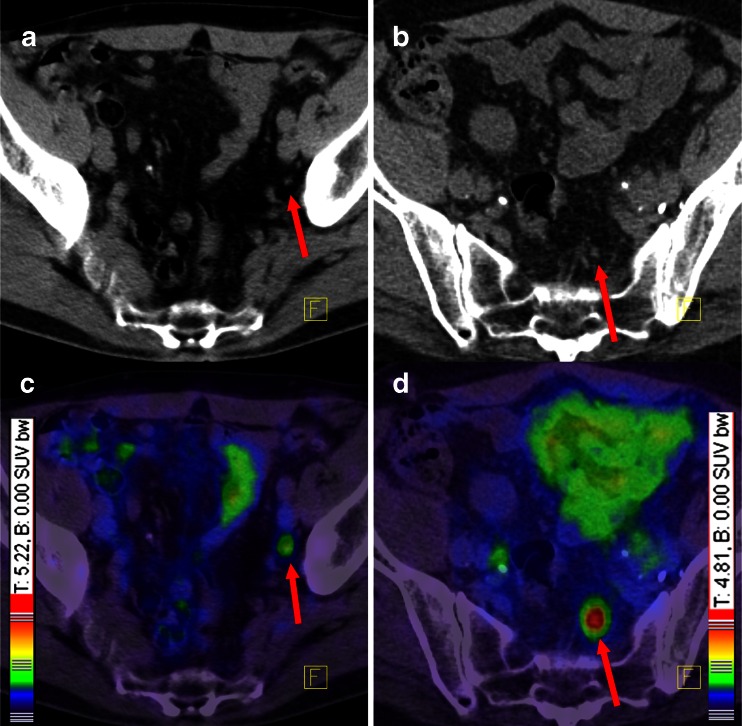
^68^Ga-PSMA-ligand PET/CT demonstrating two different patients with small lymph node metastases and different intensity of tracer uptake. Both patients had GSC 7. According to our experiences and the histological analysis, even low ^68^Ga-PSMA-HBED-CC accumulations in lesions outside the prostate gland have to be regarded as PCa-specific until proven otherwise. *Red arrows* point to lymph node metastases. Colour scales were automatically produced by the PET/CT machine. **a** CT of the first patient, **b** CT of the second patient, **c** fusion of PET and CT of the first patient, **d** fusion of PET and CT of the second patient

**Fig. 2:**
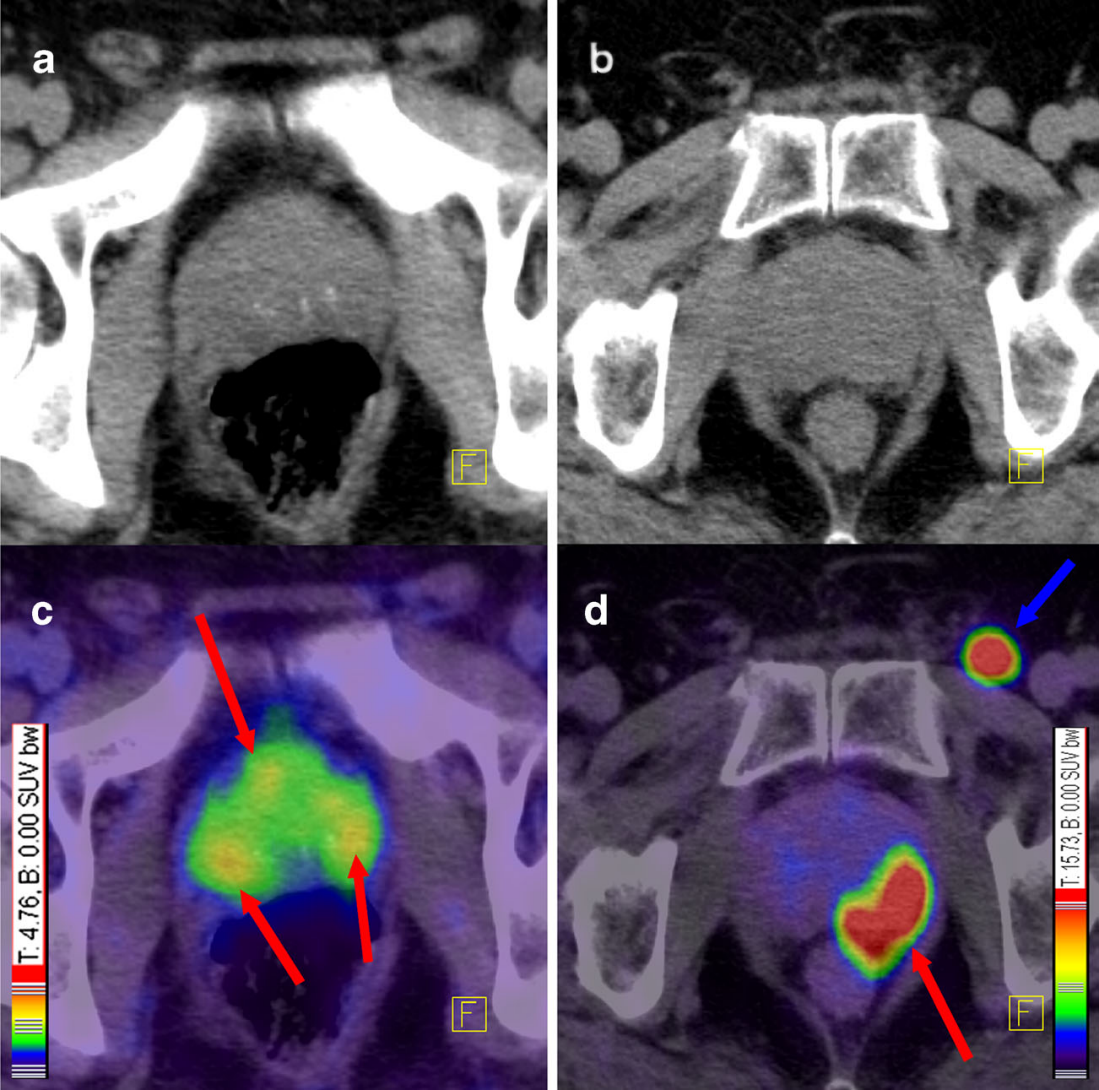
A patient with multifocal PCa (**a**, **c**) and another patient (**b**, **d**) with unifocal PCa with a rarely seen inguinal lymph node metastasis. *Red arrows* point to PCa within the prostate gland and *blue arrow* points to an inguinal lymph node metastasis. Both patients had GSC 7, although the tumours present with different contrast. Colour scales were automatically produced by the PET/CT machine. **a** Low-dose CT of the patient with a multifocal PCa, **c** corresponding fusion of PET and low-dose CT 1 h p.i., **b** low-dose CT of the patients with the unifocal PCa, **d** corresponding fusion of PET and low-dose CT 1 h p.i.

**Fig. 3:**
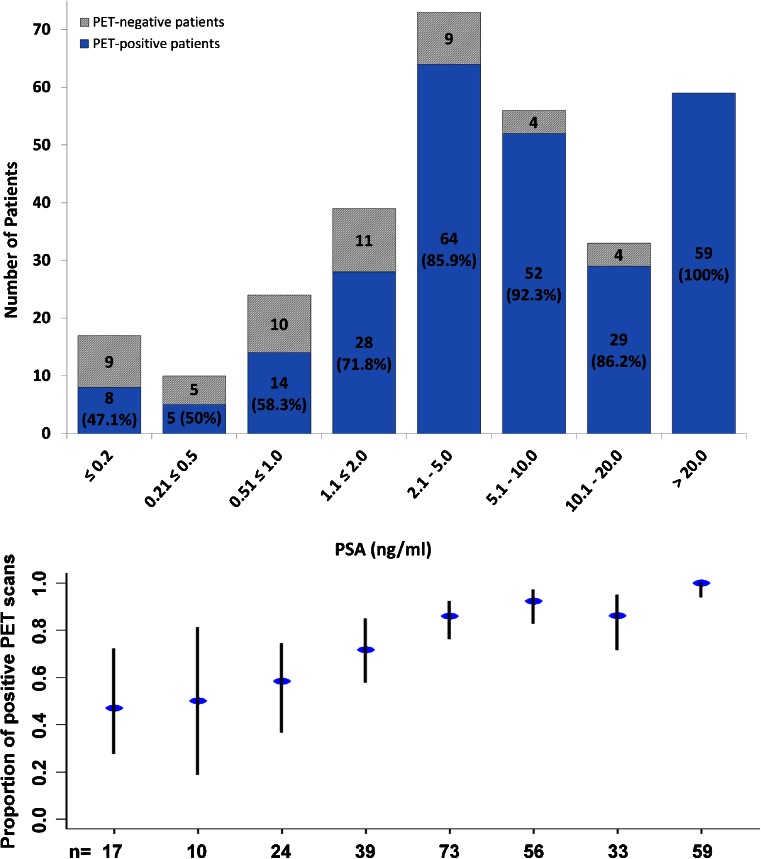
Probability of a pathological ^68^Ga-PSMA-ligand PET/CT as histogram (*above*) and plot of the rates of pathological PET/CTs with confidence intervals (*below*) depending on PSA levels in 311 patients. *Blue columns* include the number of pathological PET/CTs and their rate in %

**Fig. 4:**
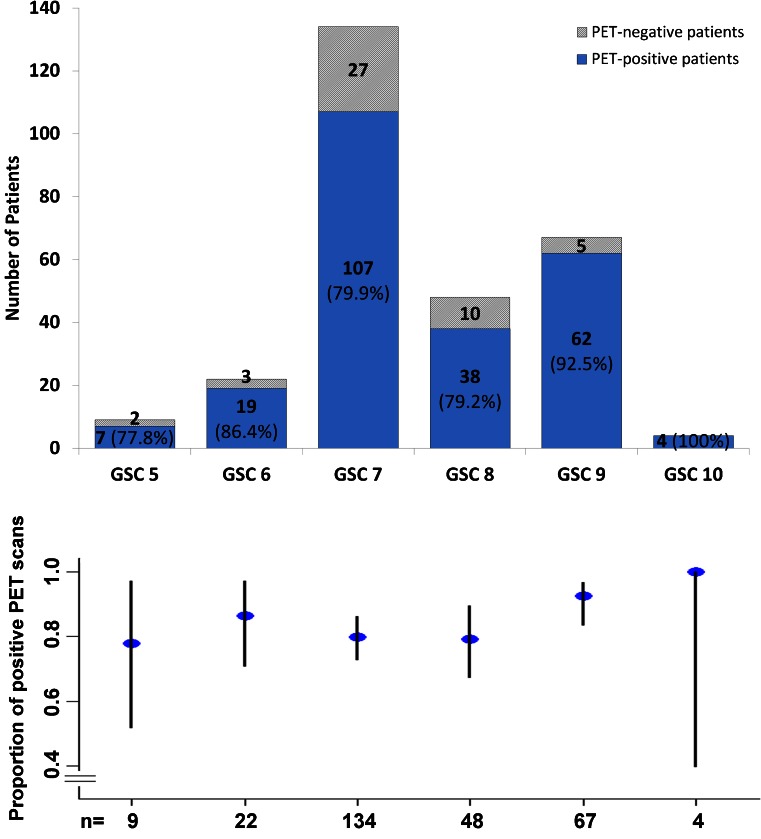
Probability of a pathological ^68^Ga-PSMA-ligand PET/CT as histogram (*above*) and plot of the rates of pathological PET/CTs with confidence intervals (*below*) depending on GSC in 284 patients. *Blue columns* include the number of pathological PET/CTs and their rate in %

There were no adverse or clinically detectable pharmacological effects in any of the patients following injection of the radiotracer. In 264 of 319 patients (82.8 %) at least 1 lesion characteristic for PCa was detected in ^68^Ga-PSMA-ligand PET/CT. Figures 1 and 2 show lymph node metastases as well as unifocal and multifocal primary PCa. Figures 3 and 4 demonstrate the probability of a pathological ^68^Ga-PSMA-ligand PET/CT depending on PSA level (311 patients) and GSC (284 patients).

Patients with pathological radiotracer uptake (*n* = 264) had a median PSA of 6.02 ng/ml (range 0.01–41,395 ng/ml), a median GSC of 7.0 (range 5–10) and were injected with a mean activity of 165.2 MBq ^68^Ga-PSMA-HBED-CC (range 40–388 MBq, median 152.5 MBq). Patients without pathological findings (*n* = 55) had a median PSA of 1.14 ng/ml (range 0.03–15.8 ng/ml), a mean GSC of 7.28 (range 5–9, median 7.0) and were injected with a mean of 180.0 ± 82.6 MBq radiotracer (range 66–400 MBq, median 160.0 MBq).

In the univariate analysis no significant difference was found between PET-positive and PET-negative patients with regard to the injected amount of ^68^Ga-PSMA-HBED-CC (logistic regression *p* = 0.165) and GSC (*t* test *p* = 0.063). However, concerning log PSA a significant difference was found (logistic regression *p* < 0.001).

Considering only the subgroup of the most challenging patients (those with PSA <0.2 ng/ml, Supplementary Table 3), no significant difference was found regarding either the injected amount of ^68^Ga-PSMA-HBED-CC or the log PSA values, while GSC was significantly higher (*t* test *p* = 0.004) in the group with pathological ^68^Ga-PSMA-ligand PET/CT.

In the multivariate analysis, a strong and significant association was detected between a positive PET result and the following parameters: log PSA level and ADT. No significant or relevant association was detected between a positive PET result and the following parameters: GSC 7, GSC 8, GSC 9–10 (all vs GSC 5–6), age and any of the defined PSA DTs. A noticeable but nonsignificant positive association was also detected between a positive ^68^Ga-PSMA-ligand PET/CT and lower amounts of injected radioactivity. The univariate analysis demonstrated no principal shifting with regard to the effect of the single variables. All uni- and multivariate values are listed in Supplementary Table 2.

SUV_max_ values of 901 representative tumour lesions were as listed in Table 2. Of the lesions, 13 were defined as local relapses after prostatectomy, 328 as lymph node metastases, 129 as soft tissue metastases, 359 as bone metastases and 72 as vital tumour lesions within the prostate gland, no matter whether they were pretreated or not. Included in the cohort of 72 lesions within the prostate gland are: (1) cases in which PET/CT was conducted before initiation of local therapy to exclude metastases after PCa was confirmed by biopsy, (2) patients who were pretreated by radiation therapy without surgery (we decided to list these patients in the “72 group” as it remains unclear if the primary tumours in PET/CT were relapses or residual vitality in tumours after radiation) and (3) patients who were pretreated by ADT only.

**Table 2:** Average SUV_max_ of all types of PCa lesions

	SUV_max_ (± SD)	Min.	Max.	Median
All Tumor Lesions (*n*=901)	13.4 (±14.6)	0.7	122.5	8.1
Local Relapse (*n*=13)	11.3 (±6.6)	2.4	22.8	11.3
Lymph Node Metastases (*n*=328)	14.2 (±17.1)	1.0	122.5	7.0
Soft Tissue Metastases (*n*=129)	8.4 (±8.0)	0.7	47.5	6.0
Bone Metastases (*n*=359)	14.3 (±14.0)	1.6	115.4	9.8
Primary PCa (*n*=72)	11.0 (±9.0)	3	40	7.1

*SD* standard deviation

Forty-two patients with pathological radiotracer uptake in ^68^Ga-PSMA-ligand PET/CT were further investigated by biopsy or surgery at our hospital. The results are listed in Table 3. In this analysis, 1 local relapse in 1 patient and 29 lymph nodes in 3 other patients were false-negative. All other tissues/lesions were true-positive (*n* = 98) or true-negative (*n* = 318). With regard to patients 34 and 37 in Table 3, PET-positive lymph nodes led to lymph node dissection of the pelvis where additional small lymph node metastases were found in the same region.

**Table 3:** Summary of histological investigations of 42 patients: 1 local relapse in 1 patient and 29 lymph node metastases in 3 other patients were false-negative. All other lesions (*n* = 416) were true-positive or true-negative

All Lesions:	False-Negative	False-Positive	True-Negative	True-Positive
Prostate Cancer	0	0	2	15
Soft Tissue Met.	0	0	4	11
Lymph Node Met.	29	0	310	65
Local Relapse	1	0	2	7
Lesion-based NPV and PPV	30/348 (8.6 %)	0/98 (0 %)	318/348 (91.4 % NPV)	98/98 (100 % PPV)
Lesion-based Specificity and Sensitivity	30/128 (23.4 %)	0/318 (0 %)	318/318 (100 % Specificity)	98/128 (76.6 % Sensitivity)
Patients:	False-Negative	False-Positive	True-Negative	True-Positive
1	0	0	0	1 (PCa)
2	0	0	1 (LN)	1 (LN)
3	0	0	2 (LN)	1 (LN)
4	0	0	9 (LN)	3 (ST)
5	0	0	15 (LN)	1+28 (ST/LN)
6	0	0	11 (LN)	1 (ST)
7	0	0	1+31 (ST/LN)	1+1 (ST/LN)
8	0	0	1 (ST)	2 (ST)
9	0	0	1 (LR)	0
10	0	0	11 (LN)	1 (PCa)
11	0	0	42 (LN)	1 (PCa)
12	0	0	12 (LN)	1 (PCa)
13	0	0	1 (PCa)	0
14	0	0	17 (LN)	1 (PCa)
15	0	0	14 (LN)	1 (PCa)
16	0	0	13 (LN)	1 (PCa)
17	0	0	0	1 (PCa)
18	0	0	37 (LN)	1 (LR)
19	0	0	0	1 (LR)
20	0	0	3 (LN)	1 (LR)
21	0	0	0	7 (LN)
22	0	0	0	1 (ST)
23	0	0	0	1 (PCa)
24	0	0	0	1 (PCa)
25	0	0	7 (LN)	1+2+2 (PCa/ST/LN)
26	1 (LN)	0	0	1 (PCa)
27	0	0	0	1 (PCa)
28	0	0	0	1 (LR)
29	0	0	24 (LN)	5+1 (LN/LR)
30	0	0	1 (LR)	0
31	1 (LR)	0	0	0
32	0	0	0	1 (LN)
33	0	0	0	1 (PCa)
34	26 (LN)	0	1+32 (ST/LN)	7 (LN)
35	0	0	1+16 (ST/LN)	1 (LN)
36	0	0	8 (LN)	1+1 (LR/LN)
37	2 (LN)	0	0	4 (LN)
38	0	0	1 (PCa)	0
39	0	0	0	1 (PCa)
40	0	0	0	1 (PCa)
41	0	0	2 (LN)	5 (LN)
42	0	0	3 (LN)	1 (LR)
Patient-based Analysis^a^	5/42 (11.9 %)	not existing	not existing	37/42 (88.1 % Sensitivity)^a^

The false-negative LN metastases of patients 35 and 37 were located in the same region as the PET-positive LNs

*PCa* prostate cancer within the prostate gland, *LN* lymph node (metastasis), *ST* soft tissue (metastasis), *LR* local relapse of PCa

^a^As described in the “Materials and methods” section, a patient-based analysis of specificity, NPV and PPV is not possible

The lesion-based analysis of sensitivity, specificity, NPV and PPV revealed values of 76.6, 100, 91.4 and 100 % (Table 3). The patient-based analysis revealed a sensitivity of 88.1 %. As described in the “Materials and methods” section, the latter value needs to be interpreted with caution and a patient-based calculation of specificity, NPV and PPV was not possible.

Follow-up information was available for 116 patients up to the time this manuscript was submitted. Amongst these, 50 received local treatment: 27 were treated with external radiation of PSMA-positive lesions (17 patients thereafter demonstrated a decrease of PSA and in 10 patients PSA was not available), 19 were operated and 4 were treated by high-intensity focused ultrasound (all presented with a decrease of PSA thereafter). Overall, 34 patients were treated by systemic therapy with ^131^I- or ^177^Lu-labelled PSMA ligands (^131^I-MIP1095 and the novel promising theranostic agent ^177^Lu-DKFZ-PSMA-617 [21, 27]). All other patients (*n* = 36) were treated by ADT and/or chemotherapy.

**Fig. 5:**
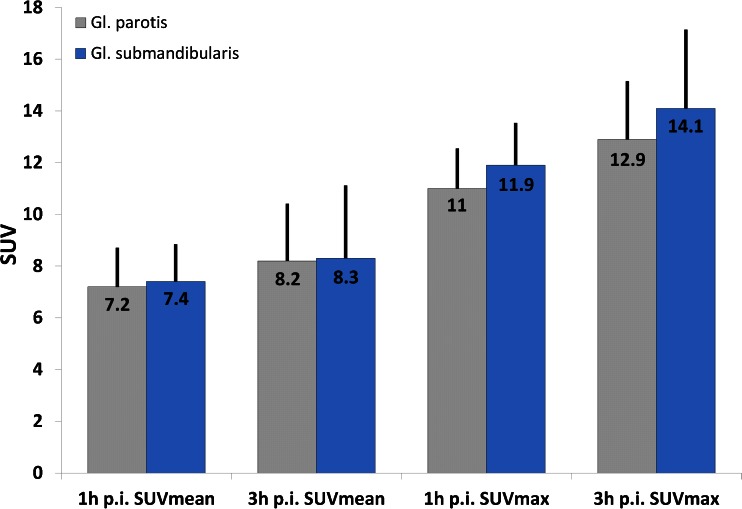
Average SUV_mean_/SUV_max_ values of ten patients who received excretion stimulus between the first ^68^Ga-PSMA-ligand PET/CT (1 h p.i.) and the second PET/CT (3 h p.i.). As demonstrated, the excretion stimulus did not reduce the high physiological radiotracer uptake of the salivary glands

Intraindividual changes in SUV_mean_/SUV_max_ values between 1 and 3 h p.i. were measured in the salivary glands of ten patients. Between both scans, an excretion stimulus using vitamin C was orally administered every 30 min as described in the “Materials and methods” section. The SUV values as well as SUV changes between 1 and 3 h p.i. (Fig. 5) were similar to our previous evaluation in which no excretion stimulus was administered [19].

## Discussion

Since the invention of the radiotracer Glu-NH-CO-NH-Lys-(Ahx)-[^68^Ga(HBED-CC)] and its clinical introduction for PET imaging at our institute in May 2011, this highly promising method (^68^Ga-PSMA-ligand PET/CT) has rapidly spread. The first clinical experiences led to the assumption that ^68^Ga-PSMA-ligand PET/CT could be a significant step forward in the diagnosis of recurrent PCa [18–20, 26]. This imaging modality is based on the observation that almost all adenocarcinomas of the prostate gland express PSMA to which Glu-NH-CO-NH-Lys-(Ahx)-[^68^Ga(HBED-CC)] is able to bind with very high affinity [28, 29]. The aim of this evaluation was to assess 319 patients who were referred to our hospital for ^68^Ga-PSMA-ligand PET/CT from the first clinical implementation of this method in May 2011 until January 2014.

Overall, 82.8 % of the patients presented with at least one lesion characteristic for PCa in ^68^Ga-PSMA-ligand PET/CT. This rate is slightly lower compared to our previous reports [19, 20]. A possible explanation is that our previous studies were conducted in the initial phase of imaging with this novel method and that after ^68^Ga-PSMA-ligand PET/CT became increasingly better known, more challenging cases with negative alternative imaging results and low PSA values were referred to our hospital.

In the multivariate analysis a strong association was detected between a pathological ^68^Ga-PSMA-ligand PET/CT and the PSA level. This result was expected as an increase in PSA levels usually indicates progressive disease. However, as demonstrated by Fig. 3, a continuous increase in PSA level does not automatically correlate with an increase in tumor detection. In general the question remains why not more patients show a pathologic ^68^Ga-PSMA-ligand PET/CT although according to literature almost all prostatic adenocarcinomas express PSMA [28, 29]. Possible reasons could be the individual tumor heterogeneity, a dedifferentiation of PCa leading to a decoupling of tumor mass and PSA level, absent or very low PSMA expression of some tumors (e.g. neuroendocrine PCa), tumors adjacent to the urinary bladder, small tumour size below the spatial resolution of the PET scanner or the stage of technology of the PET scanner. With regard to the latter aspect we believe for example that new scanners with time of flight (TOF) technology enable more sensitive tumour detection.

In the most challenging cohort (patients with PSA <0.2 ng/ml) GSC was significantly higher in the group with pathological ^68^Ga-PSMA-ligand PET/CT compared to the group without pathological ^68^Ga-PSMA-ligand PET/CT. However, with regard to all patients analysed in this evaluation, the multivariate analysis did not show a relevant association between a positive ^68^Ga-PSMA-ligand PET/CT and GSC 7, 8 or 9–10 compared to a low GSC of 5–6. This result is contrary to our expectations: in the literature, a positive correlation between higher GSC and PSMA expression has been demonstrated in preclinical studies [30–32]. The sole explanation we have at this stage is that the small cohort of patients with GSC 5–6 causes substantial variability in the statistical analysis. We consider GSC as an important variable in both imaging and therapy with PSMA ligands. Further studies focusing on this topic are eagerly awaited.

The multivariate analysis also did not demonstrate a relevant association between a positive ^68^Ga-PSMA-ligand PET/CT and the five defined PSA DTs. At this stage, we assume that there is no significant correlation between proliferation index and PSMA expression of the tumours.

Patients with an ADT at the time of ^68^Ga-PSMA-ligand PET/CT more frequently showed a positive PET scan compared to patients without such treatment. One explanation for this result could be that patients with advanced disease are more often referred to ADT. However, this theory needs to be investigated in more detail as many cases exist in which ADT is started at low PSA values. We believe that the time to start ADT depends on many variables such as patients’ demands, preferences/experience of physicians, PSA DT, etc. In addition, ADT usually leads to a reduction of PSA levels and can also reduce the tumour size [33, 34]. Both have the potential to negatively influence the detection rate in imaging modalities. Another explanation for our findings could be that the PSMA expression is apparently differentially regulated by androgens. In previous preclinical studies, PSMA expression was downregulated by androgen therapy and upregulated by antiandrogen therapy [35–37]. This counterintuitive behaviour is not yet fully understood and more studies are eagerly awaited to investigate the influence of ADT on both diagnosis and therapy with PSMA ligands.

A noticeable but nonsignificant positive association was also detected between a positive ^68^Ga-PSMA-ligand PET/CT and lower amounts of injected radioactivity. However, according to our experience in routine clinical practice, tracer activities of more than 100–120 MBq lead to improved image quality while especially amounts less than 60–70 MBq cause the opposite. At this stage, our sole explanation for this unexpected finding is statistical variability. We do not believe that higher radioactivity amounts lead to any adverse molecular interaction as all injections contained 2 nmol PSMA ligand.

The analysis of SUV values within the manifestations of PCa demonstrate that uptake of ^68^Ga-PSMA-HBED-CC is high in primary tumours as well as in local relapses and all types of metastases. This result confirms our previous evaluation in which we could demonstrate that the total uptake of PCa lesions as well as tumour contrast are significantly higher compared to choline PET/CT [20]. However, as shown in the aforementioned evaluation, it has to be considered that lymph node metastases usually present with the highest contrast, followed by bone metastases, local relapses and soft tissue metastases.

We assume that virtually all patients referred to our hospital had recurrent disease or persistent primary tumour and that our cohort did not include true-negative cases. Therefore, calculating patient-based specificity, NPV and PPV is not possible as described in the “Materials and methods” section. However, with regard to a lesion-based analysis, it is apparent that ^68^Ga-PSMA-HBED-CC is able to detect PCa with good sensitivity and excellent specificity (Table 3). Only 1 local relapse in 1 patient and 29 lymph node metastases in 3 other patients were false-negative. With regard to two of the latter patients (patients 34 and 37 in Table 3), PET-positive lymph nodes led to lymph node dissection of the pelvis where additional small lymph node metastases were found. These PET-negative lymph nodes were located in the same region as the PET-positive lymph nodes and had therefore no negative influence on the therapy procedure. We assume that the mentioned metastases were not detected by ^68^Ga-PSMA-ligand PET/CT due to a small size and low PSMA expression. However, with the exception of the above-mentioned PET-negative metastases in four different patients, all other lesions (*n* = 416) were true-positive or true-negative. This result demonstrates that ^68^Ga-PSMA-HBED-CC is highly specific for PCa. However, theoretically an expression of PSMA within inflammatory lesions can exist, although it is considered to be rare [29].

Overall, the histology results need to be interpreted with caution, e.g. cases with different numbers of lymph node metastases usually cause a bias. In addition, there was no standardized protocol to investigate PET-positive lesions. Calculating patient-based values of specificity, NPV and PPV is therefore not reasonable as mentioned before. The most important message of the histology results is that ^68^Ga-PSMA-HBED-CC is highly specific for PCa. At this stage, we suggest that accumulations of ^68^Ga-PSMA-HBED-CC in lesions outside the prostate gland have to be regarded as PCa-specific until proven otherwise. In addition, we suggest that specification of a cut-off SUV for malignant or benign tissue is not reasonable for lesions outside of the prostate gland. On the other hand, for lesions within the prostate gland, calculation of such cut-off SUVs can possibly help to distinguish different GSC or tumour and inflammatory tissue. However, such cut-offs cannot be provided by this evaluation as a sufficient number of benign tissues need to be analysed. Stereotactic biopsy studies are underway to provide more data.

Among the 116 patients who were available for follow-up, 50 (40 %) were treated locally after ^68^Ga-PSMA-ligand PET/CT and therefore delayed systemic therapy due to the results of the ^68^Ga-PSMA-ligand PET/CT. We believe that such patients who can delay systemic therapy have a greater potential for improved quality of life. Systemic therapies usually present with significant side effects and the therapeutic effect is frequently temporary. It also has to be considered that patients who present with locally accessible tumours have theoretically a better chance to be cured of their disease. However, long-term studies are necessary to evaluate the impact of ^68^Ga-PSMA-ligand PET/CT on the overall survival time and quality of life of PCa patients.

In this evaluation we could also demonstrate that a sour excretion stimulus does not reduce the high physiological radiotracer uptake of the salivary glands. We therefore assume that such a stimulus would not help to reduce radiation-related side effects of therapy with radiolabelled PSMA ligands.

Our retrospective analysis shows typical limitations compared to prospective studies: biased patient referrals and thereby possibly biased treatments and follow-up as well. However, we believe that our data reflect the daily clinical routine and that every centre which conducts ^68^Ga-PSMA-ligand PET/CT faces similar conditions. Our results and messages therefore can help to better understand PSMA imaging until more data are available in the future.

### Conclusion

This evaluation investigated the diagnostic value of PET/CT imaging with the new ^68^Ga-labelled PSMA ligand HBED-CC (^68^Ga-DKFZ-PSMA-11) in the diagnosis of PCa. ^68^Ga-PSMA-ligand PET/CT 1 h p.i. can detect PCa in a high percentage of patients with suspected cancer (82.8 %). In addition, the tracer is highly specific for PCa: histological analysis demonstrated that accumulation of ^68^Ga-PSMA-HBED-CC in lesions correlates with manifestations of PCa in virtually all cases and false-positive lesions could not be detected. Detection of PCa is improved at higher PSA levels. Also, ADT had a significantly positive influence on tumour detection rate, which, however, has to be interpreted with caution. However, no association was found between a positive PET/CT and faster PSA DTs as well as GSC 7, 8 or 9–10 compared to a low GSC of 5–6. Amongst all patients who were available for follow-up after ^68^Ga-PSMA-ligand PET/CT, 40 % could be treated locally with resulting delayed systemic therapy. We could also demonstrate that an excretion stimulus using vitamin C does not lead to a reduction of the physiologically high ^68^Ga-PSMA-HBED-CC uptake in salivary glands. With regard to possible therapy of metastatic PCa with radiolabelled PSMA ligands, we therefore assume that such an excretion stimulus would not help to reduce radiation-based off-target effects to the salivary glands.

## Electronic supplementary material

Below is the link to the electronic supplementary material.

Supplementary Table 1 Patients’ characteristics. (PDF 429 kb)

Supplementary Table 2 Part A (*upper table*): results of the univariate and multivariate analyses for several variables possibly interacting with ^68^Ga-PSMA PET/CT. The number of patients for whom all listed variables are available is 277. Part B (*lower table*): same analyses as above but extended by several PSA doubling times (PSA DT). By including the PSA DT, the number of patients for whom all listed variables are available drops to 171. *ADT* androgen deprivation therapy, *GSC* Gleason score. (GIF 318 kb)

High-resolution image (TIF 2.64 mb)

Supplementary Table 3 Characteristics of patients with PSA values < 0.2 ng/ml. (GIF 123 kb)

High-resolution image (TIF 551 kb)

## References

[1] Siegel R, Ma J, Zou Z, Jemal A (2014). Cancer statistics, 2014. CA Cancer J Clin.

[2] Aus G, Abbou CC, Bolla M, Heidenreich A, Schmid HP, Van Poppel H (2005). EAU guidelines on prostate cancer. Eur Urol.

[3] Kosuri S, Akhtar NH, Smith M, Osborne JR, Tagawa ST. Review of salvage therapy for biochemically recurrent prostate cancer: the role of imaging and rationale for systemic salvage targeted anti-prostate-specific membrane antigen radioimmunotherapy. Adv Urol 2012;2012:921674. doi:10.1155/2012/921674. 10.1155/2012/921674 PMC3368159 22693495

[4] Schmid DT, John H, Zweifel R, Cservenyak T, Westera G, Goerres GW (2005). Fluorocholine PET/CT in patients with prostate cancer: initial experience. Radiology.

[5] Igerc I, Kohlfürst S, Gallowitsch HJ, Matschnig S, Kresnik E, Gomez-Segovia I (2008). The value of 18F-choline PET/CT in patients with elevated PSA-level and negative prostate needle biopsy for localisation of prostate cancer. Eur J Nucl Med Mol Imaging.

[6] Kwee SA, DeGrado T (2008). Prostate biopsy guided by 18F-fluorocholine PET in men with persistently elevated PSA levels. Eur J Nucl Med Mol Imaging.

[7] Häcker A, Jeschke S, Leeb K, Prammer K, Ziegerhofer J, Sega W (2006). Detection of pelvic lymph node metastases in patients with clinically localized prostate cancer: comparison of [18F]fluorocholine positron emission tomography-computerized tomography and laparoscopic radioisotope guided sentinel lymph node dissection. J Urol.

[8] Husarik DB, Miralbell R, Dubs M, John H, Giger OT, Gelet A (2008). Evaluation of [(18)F]-choline PET/CT for staging and restaging of prostate cancer. Eur J Nucl Med Mol Imaging.

[9] Hillier SM, Maresca KP, Femia FJ, Marquis JC, Foss CA, Nguyen N, et al. Preclinical evaluation of novel glutamate-urea-lysine analogues that target prostate-specific membrane antigen as molecular imaging pharmaceuticals for prostate cancer. Cancer Res 2009;69:6932–40. doi:10.1158/0008-5472.CAN-09-1682. 10.1158/0008-5472.CAN-09-1682 PMC4114247 19706750

[10] Eder M, Schäfer M, Bauder-Wüst U, Hull WE, Wängler C, Mier W, et al. 68Ga-complex lipophilicity and the targeting property of a urea-based PSMA inhibitor for PET imaging. Bioconjug Chem 2012;23:688–97. doi:10.1021/bc200279b. 10.1021/bc200279b 22369515

[11] Schäfer M, Bauder-Wüst U, Leotta K, Zoller F, Mier W, Haberkorn U, et al. A dimerized urea-based inhibitor of the prostate-specific membrane antigen for 68Ga-PET imaging of prostate cancer. EJNMMI Res 2012;2:23. doi:10.1186/2191-219X-2-23. 10.1186/2191-219X-2-23 PMC3502552 22673157

[12] Bander NH (2006). Technology insight: monoclonal antibody imaging of prostate cancer. Nat Clin Pract Urol.

[13] Liu H, Moy P, Kim S, Xia Y, Rajasekaran A, Navarro V (1997). Monoclonal antibodies to the extracellular domain of prostate-specific membrane antigen also react with tumor vascular endothelium. Cancer Res.

[14] Sweat SD, Pacelli A, Murphy GP, Bostwick DG (1998). Prostate-specific membrane antigen expression is greatest in prostate adenocarcinoma and lymph node metastases. Urology.

[15] Mannweiler S, Amersdorfer P, Trajanoski S, Terrett JA, King D, Mehes G (2009). Heterogeneity of prostate-specific membrane antigen (PSMA) expression in prostate carcinoma with distant metastasis. Pathol Oncol Res.

[16] Chen Y, Pullambhatla M, Foss CA, Byun Y, Nimmagadda S, Senthamizhchelvan S, et al. 2-(3-{1-Carboxy-5-[(6-[18F]fluoro-pyridine-3-carbonyl)-amino]-pentyl}-ureido)-pen tanedioic acid, [18F]DCFPyL, a PSMA-based PET imaging agent for prostate cancer. Clin Cancer Res 2011;17:7645–53. doi:10.1158/1078-0432.CCR-11-1357. 10.1158/1078-0432.CCR-11-1357 PMC3243762 22042970

[17] Eder M, Eisenhut M, Babich J, Haberkorn U. PSMA as a target for radiolabelled small molecules. Eur J Nucl Med Mol Imaging 2013;40:819–23. doi:10.1007/s00259-013-2374-2. 10.1007/s00259-013-2374-2 PMC3644196 23463331

[18] Afshar-Oromieh A, Haberkorn U, Schlemmer HP, Fenchel M, Eder M, Eisenhut M, et al. Comparison of PET/CT and PET/MRI hybrid systems using a (68)Ga-labelled PSMA ligand for the diagnosis of recurrent prostate cancer: initial experience. Eur J Nucl Med Mol Imaging 2014;41:887–97. doi:10.1007/s00259-013-2660-z. 10.1007/s00259-013-2660-z 24352789

[19] Afshar-Oromieh A, Malcher A, Eder M, Eisenhut M, Linhart HG, Hadaschik BA, et al. PET imaging with a [68Ga]gallium-labelled PSMA ligand for the diagnosis of prostate cancer: biodistribution in humans and first evaluation of tumour lesions. Eur J Nucl Med Mol Imaging 2013;40:486–95. doi:10.1007/s00259-012-2298-2. 10.1007/s00259-012-2298-2 23179945

[20] Afshar-Oromieh A, Zechmann CM, Malcher A, Eder M, Eisenhut M, Linhart HG, et al. Comparison of PET imaging with a (68)Ga-labelled PSMA ligand and (18)F-choline-based PET/CT for the diagnosis of recurrent prostate cancer. Eur J Nucl Med Mol Imaging 2014;41:11–20. doi:10.1007/s00259-013-2525-5. 10.1007/s00259-013-2525-5 PMC3843747 24072344

[21] Zechmann CM, Afshar-Oromieh A, Armor T, Stubbs JB, Mier W, Hadaschik B, et al. Radiation dosimetry and first therapy results with a (124)I/(131)I-labeled small molecule (MIP-1095) targeting PSMA for prostate cancer therapy. Eur J Nucl Med Mol Imaging 2014;41:1280–92. doi:10.1007/s00259-014-2713-y. 10.1007/s00259-014-2713-y PMC4052014 24577951

[22] Lapi SE, Wahnishe H, Pham D, Wu LY, Nedrow-Byers JR, Liu T (2009). Assessment of an 18F-labeled phosphoramidate peptidomimetic as a new prostate-specific membrane antigen-targeted imaging agent for prostate cancer. J Nucl Med.

[23] Holland JP, Divilov V, Bander NH, Smith-Jones PM, Larson SM, Lewis JS. 89Zr-DFO-J591 for immunoPET of prostate-specific membrane antigen expression in vivo. J Nucl Med 2010;51:1293–300. doi:10.2967/jnumed.110.076174. 10.2967/jnumed.110.076174 PMC2998794 20660376

[24] Afshar-Oromieh A, Haberkorn U, Eder M, Eisenhut M, Zechmann CM. [68Ga]Gallium-labelled PSMA ligand as superior PET tracer for the diagnosis of prostate cancer: comparison with 18F-FECH. Eur J Nucl Med Mol Imaging 2012;39:1085–6. doi:10.1007/s00259-012-2069-0. 10.1007/s00259-012-2069-0 22310854

[25] Afshar-Oromieh A, Haberkorn U, Hadaschik B, Habl G, Eder M, Eisenhut M, et al. PET/MRI with a 68Ga-PSMA ligand for the detection of prostate cancer. Eur J Nucl Med Mol Imaging 2013;40:1629–30. doi:10.1007/s00259-013-2489-5. 10.1007/s00259-013-2489-5 23817686

[26] Roethke MC, Kuru TH, Afshar-Oromieh A, Schlemmer HP, Hadaschik BA, Fenchel M. Hybrid positron emission tomography-magnetic resonance imaging with gallium 68 prostate-specific membrane antigen tracer: a next step for imaging of recurrent prostate cancer-preliminary results. Eur Urol 2013;64:862–4. doi:10.1016/j.eururo.2013.08.003. 10.1016/j.eururo.2013.08.003 23954084

[27] Benesova M, Schäfer M, Bauder-Wüst U, Mier W, Haberkorn U, Eisenhut M (2013). Linker modifications of DOTA-conjugated inhibitors of the prostate-specific membrane antigen (PSMA). Eur J Nucl Med Mol Imaging.

[28] Ross JS, Sheehan CE, Fisher HA, Kaufman RP, Kaur P, Gray K (2003). Correlation of primary tumor prostate-specific membrane antigen expression with disease recurrence in prostate cancer. Clin Cancer Res.

[29] Silver DA, Pellicer I, Fair WR, Heston WD, Cordon-Cardo C (1997). Prostate-specific membrane antigen expression in normal and malignant human tissues. Clin Cancer Res.

[30] Marchal C, Redondo M, Padilla M, Caballero J, Rodrigo I, García J (2004). Expression of prostate specific membrane antigen (PSMA) in prostatic adenocarcinoma and prostatic intraepithelial neoplasia. Histol Histopathol.

[31] Kasperzyk JL, Finn SP, Flavin R, Fiorentino M, Lis R, Hendrickson WK, et al. Prostate-specific membrane antigen protein expression in tumor tissue and risk of lethal prostate cancer. Cancer Epidemiol Biomarkers Prev 2013;22:2354–63. doi:10.1158/1055-9965.EPI-13-0668. 10.1158/1055-9965.EPI-13-0668 PMC3893763 24130224

[32] Minner S, Wittmer C, Graefen M, Salomon G, Steuber T, Haese A, et al. High level PSMA expression is associated with early PSA recurrence in surgically treated prostate cancer. Prostate 2011;71:281–8. doi:10.1002/pros.21241. 10.1002/pros.21241 20809553

[33] Lilleby W, Fosså SD, Knutsen BH, Abildgaard A, Skovlund E, Lien HH (2000). Computed tomography/magnetic resonance based volume changes of the primary tumour in patients with prostate cancer with or without androgen deprivation. Radiother Oncol.

[34] Resnick MI (1984). Hormonal therapy in prostatic carcinoma. Urology.

[35] Wright GL, Grob BM, Haley C, Grossman K, Newhall K, Petrylak D (1996). Upregulation of prostate-specific membrane antigen after androgen-deprivation therapy. Urology.

[36] Liu T, Wu LY, Fulton MD, Johnson JM, Berkman CE. Prolonged androgen deprivation leads to downregulation of androgen receptor and prostate-specific membrane antigen in prostate cancer cells. Int J Oncol 2012;41:2087–92. doi:10.3892/ijo.2012.1649. 10.3892/ijo.2012.1649 PMC3583693 23041906

[37] Evans MJ, Smith-Jones PM, Wongvipat J, Navarro V, Kim S, Bander NH, et al. Noninvasive measurement of androgen receptor signaling with a positron-emitting radiopharmaceutical that targets prostate-specific membrane antigen. Proc Natl Acad Sci U S A 2011;108:9578–82. doi:10.1073/pnas.1106383108. 10.1073/pnas.1106383108 PMC3111331 21606347

