# Supplementary material for: The diagnostic value of PET/CT imaging with the 68Ga-labelled PSMA ligand HBED-CC in the diagnosis of recurrent prostate cancer
Source: Eur J Nucl Med Mol Imaging. 2014 Nov 20;42(2):197–209. doi: 10.1007/s00259-014-2949-6 (PMC4315487; doi:10.1007/s00259-014-2949-6)
Supplement: Supplementary file 1 — Patients’ characteristics. (PDF 429 kb) [file 259_2014_2949_MOESM1_ESM.pdf]

Supplementary Table 1 patients characteristics. Red rows indicate non-pathologic ^{68}Ga-PSMA-PET/CTs. *GSC* Gleason Score; *PSA-DT* PSA doubling- time; *PEx* prostatectomy; *Rx* radiation therapy of the prostate gland or prostate fossa after prostatectomy; *ADT* androgen deprivation therapy.

Patient no.	Age (y)	PET dd/mm/yy	Tracer (MBq)	GSC	PSA at PET ng/ml	PSA-DT (days)	PEx	TNM	Rx	ADT
1	76	24.05.2011	114	7	n/a	n/a	yes	pT2a pN0 M0	yes	no
2	79	08.06.2011	100	8	n/a	n/a	no	T2c	yes	no
3	66	15.06.2011	149	5	1.50	84	yes	pT3a pN0 M0 R1	yes	no
4	67	06.07.2011	111	7	7.40	n/a	nein	T4	yes	no
5	60	07.07.2011	100	9	5.56	240	yes	pT3 pN1 (6/25)	no	yes
6	75	19.07.2011	68	8	2.67	150	yes	pT2a pN0 R0 G3	yes	no
7	68	21.07.2011	68	7	3.20	484	yes	n/a	no	no
8	71	04.08.2011	132	7	3.20	n/a	no	T1c Nx Mx	yes	no
9	67	09.08.2011	120	7	1.13	74	yes	pT2c pN0 (0/23) R1	no	no
10	76	11.08.2011	118	7	2.63	n/a	yes	pT2c pN0 G3 R0	no	no
11	64	08.09.2011	120	8	0.03	n/a	yes	pT2c pN0/1 L0 Pn0 R1	no	no
12	73	12.09.2011	96	7	2.00	n/a	yes	pT3a N0 M0	yes	no
13	67	19.09.2011	105	9	36.00	141	yes	n/a	no	no
14	67	28.09.2011	111	9	1.20	n/a	yes	pT3b, pN1(9/45), G3, R1	yes	no
15	67	10.10.2011	52	9	1.80	n/a	yes	pT3b pN0 L1 G3	yes	no
16	70	24.10.2011	90	7	1.00	454	yes	pT3a pN0 M0 R0	yes	no
17	71	04.11.2011	116	5	5.60	72	no	pT1c	no	no
18	72	08.11.2011	139	6	7.40	n/a	no	cT2a N0 M0	yes	no
19	72	09.11.2011	177	9	3.58	134	no	n/a	yes	yes
20	64	10.11.2011	81	8	334.00	183	yes	pT3, pN1, pMx, R1	yes	no
21	73	11.11.2011	144	7	985.00	18	yes	pT2a N1 (1/15) pMx G3a	no	no
22	65	16.11.2011	123	7	n/a	n/a	yes	pT2c, pN0, M0, R0, G3	yes	yes
23	51	17.11.2011	148	6	1.20	n/a	yes	pT2b Nx M0	yes	yes
24	67	23.11.2011	68	9	9.60	n/a	yes	cT4 cN+ Mx	yes	yes
25	61	24.11.2011	152	7	0.10	n/a	yes	pT3a pN0 M1	no	no
26	74	25.11.2011	169	8	20.06	3261	yes	n/a	no	no
27	70	07.12.2011	350	9	1.06	n/a	yes	T4 N0 M0 pR1	n/a	no
28	78	08.12.2011	182	7	7.46	276	yes	pT3b, pN0, G3a	yes	no
29	85	09.12.2011	93	7	2.59	587	no	pT2c N0 M0	yes	no
30	70	13.12.2011	172	7	4.90	98	yes	pT3a, pN0 (0/4), R0	yes	no
31	73	16.12.2011	133	7	n/a	n/a	yes	pT3 N1 R1 L1	yes	no
32	55	21.12.2011	103	9	148.00	250	yes	pT3B N 1 , M1	yes	yes
33	69	22.12.2011	117	7	7.40	487	yes	pT3b pN0 R1	yes	no
34	72	10.01.2012	96	9	4.80	n/a	yes	pT3 pN0 M0	yes	yes
35	74	11.01.2012	82	8	0.04	n/a	yes	pT3a pN0 (0/7) M0	yes	no
36	66	18.01.2012	96	9	0.01	n/a	yes	pT3b pN0	no	yes
37	75	24.01.2012	52	5	11.90	47	no	n/a	yes	yes
38	71	25.01.2012	76	6	0.40	n/a	no	n/a	yes	yes
39	55	26.01.2012	44	9	9.54	n/a	yes	pT3b pN1 (6/11) R0	no	yes
40	72	26.01.2012	40	n/a	13.80	450	yes	n/a	yes	yes
41	70	27.01.2012	130	7	31.00	n/a	yes	pT3 pN0 M0 L1	yes	yes

42	56	31.01.2012	109	9	28.20	n/a	yes	cT3a cN0 M0	yes	yes
43	73	02.02.2012	218	6	122.00	15	yes	pT3 pN0 M0	yes	no
44	48	03.02.2012	165	8	n/a	n/a	yes	pT2c pN1	yes	no
45	61	07.02.2012	59	8	1.98	24	yes	pT3b pN0 R1 G3	no	no
46	65	15.02.2012	185	9	7.08	n/a	yes	cT2 N0 M1	no	yes
47	70	16.02.2012	200	7	0.46	26	yes	pT3b	yes	no
48	86	17.02.2012	119	8	2.90	207	no	T1c Nx Mx	yes	no
49	63	22.02.2012	107	9	0.10	n/a	yes	pT3a pN0 (0/10) M0	yes	no
50	72	23.02.2012	130	8	3.30	497	yes	pT2a pN0 M0	yes	no
51	65	24.02.2012	212	6	11.20	n/a	no	n/a	no	no
52	61	28.02.2012	65	7	1.72	125	yes	pT3b pN0 M0 G3	no	no
53	67	29.02.2012	148	7	0.10	n/a	yes	pT2c pN0 (0/21) cM0 G3	yes	no
54	70	02.03.2012	135	n/a	20.10	n/a	no	n/a	no	no
55	72	09.03.2012	79	8	10.60	440	yes	pT3a pN0(0/10) M0	no	no
56	58	14.03.2012	67	9	2.35	n/a	yes	pT3b pN0 L1 V0 R0	yes	no
57	68	15.03.2012	61	7	6.34	69	yes	ypT3b pN1(5/35) R0	no	no
58	81	16.03.2012	103	7	116.00	n/a	yes	pT1cG3pN0	yes	no
59	61	21.03.2012	85	9	2.22	124	yes	pT2b, N0, M0, G3	yes	no
60	81	23.03.2012	148	7	14.60	n/a	no	T1c N0 Mx	yes	no
61	75	27.03.2012	240	7	80.30	62	no	T1c N0 M0	yes	no
62	64	28.03.2012	167	9	146.00	n/a	no	T4 N2 M2	yes	yes
63	67	30.03.2012	175	8	2.81	23	yes	pT2c pN0 R0	no	no
64	62	03.04.2012	142	9	3.78	164	yes	pT4, pN1(7/25)	yes	yes
65	62	04.04.2012	161	7	0.10	n/a	yes	pT3 pN0 R1	no	no
66	67	05.04.2012	150	6	31.80	n/a	no	n/a	no	no
67	59	11.04.2012	154	9	4.21	22	yes	pT2c pN0 R0	yes	no
68	65	12.04.2012	227	7	0.48	n/a	no	n/a	no	yes
69	64	13.04.2012	232	8	0.10	n/a	yes	pT2c pN0 pR0	yes	yes
70	70	18.04.2012	79	7	2.82	144	yes	pT3b N0 M0 C61	yes	no
71	61	19.04.2012	263	8	12.50	197	yes	T2b C2 N0 M0 G3	yes	yes
72	69	20.04.2012	150	7	1.86	n/a	yes	pT2 pN1	yes	yes
73	67	24.04.2012	182	9	6.47	n/a	yes	pT3b pN1 G3 R0	yes	no
74	75	25.04.2012	110	5	12.90	n/a	no	pT2b G2	yes	yes
75	69	26.04.2012	101	7	1.59	101	yes	pT2 N1 M0 R1	yes	no
76	76	02.12.2011	132	9	100.00	n/a	yes	pT3a pN1 M0 G3 R0	no	no
77	76	04.05.2012	84	7	8.73	n/a	yes	pT3a L1 V1 G3 M0 R0	no	no
78	76	09.05.2012	215	7	41.50	n/a	yes	pT2 pN0 G2	yes	no
79	68	14.05.2012	205	9	10.10	n/a	yes	n/a	n/a	no
80	59	15.05.2012	139	9	4.90	164	no	n/a	no	yes
81	68	22.05.2012	77	7	6.59	58	yes	pT3a pN0 M0	yes	no
82	73	23.05.2012	144	10	2.46	75	no	n/a	yes	yes
83	51	24.05.2012	188	9	176.00	75	yes	n/a	no	yes
84	61	30.05.2012	76	7	4.22	124	yes	pT3b N1 R1	yes	no
85	57	01.06.2012	146	8	5.34	31	yes	pT3a pN0 R1 G3	yes	no
86	65	12.06.2012	164	7	0.30	n/a	yes	pT2c pN0 cM0 V0 Pn1 R0	no	no
87	67	14.06.2012	127	7	6.95	19	yes	pT2a,pN0 (0/13), R0	yes	no
88	74	15.06.2012	130	8	61.30	n/a	yes	pT3a Nx cM0 R1	no	no

89	84	19.06.2012	99	n/a	2.61	n/a	no	pT2 pN0 M0 G1	yes	yes
90	62	21.06.2012	88	7	1.15	419	yes	pT2c pN0 (0/) L0 V0 R0 G2	yes	no
91	69	22.06.2012	180	7	60.30	49	yes	R1 N+ (4/xx)	yes	yes
92	61	26.06.2012	138	7	2.24	39	yes	pT3 pN0 G3	yes	yes
93	56	27.06.2012	172	7	0.91	190	yes	pT3b pN0	yes	no
94	58	28.06.2012	166	7	1.34	n/a	yes	n/a	no	no
95	71	26.06.2012	105	9	28.40	109	yes	pT3b pN1R1	no	yes
96	66	03.07.2012	111	10	139.00	55	yes	pT3b pN1 pL1 pV0 R1	no	yes
97	65	03.07.2012	257	9	0.10	n/a	yes	pT3b, pN1 (10/27) , R1	no	yes
98	59	04.07.2012	257	9	4.14	93	yes	pT3 N1 M0	no	no
99	74	06.07.2012	76	7	23.38	89	yes	pT2c pN0 pMx R0	yes	yes
100	66	09.07.2012	151	8	12.70	2707	no	n/a	no	no
101	70	09.07.2012	194	7	283.00	n/a	yes	pT3b pN1 (1/25) M0 R1	yes	no
102	62	10.07.2012	104	7	3.17	59	yes	pT3b pN1 (1/19) M0 L1	yes	no
103	72	11.07.2012	285	n/a	33.00	n/a	yes	pT3b N0 M0 R1	yes	yes
104	71	12.07.2012	163	9	43.50	n/a	yes	cT4	yes	no
105	70	13.07.2012	150	8	258.00	36	yes	pT2c pN0 R1	yes	yes
106	65	16.07.2012	159	10	7.01	1526	no	n/a	no	no
107	69	16.07.2012	141	7	0.76	893	yes	pT2 pN0 (0/22) M0	no	no
108	74	17.07.2012	106	7	4.98	n/a	no	T1c G3	no	yes
109	63	19.07.2012	160	7	13.00	24	yes	pT3b N0	yes	no
110	63	26.07.2012	220	9	265.00	n/a	no	n/a	no	yes
111	57	31.07.2012	135	7	8.30	n/a	yes	pT3b pN0 R0	no	no
112	60	01.08.2012	136	7	10.30	40	no	T2a NX MX	yes	no
113	57	06.08.2012	122	7	9.12	n/a	yes	cT3a	n/a	yes
114	63	06.08.2012	118	7	5.60	118	yes	pT3a pN0 M0	yes	no
115	56	06.08.2012	133	7	1.69	66	yes	pT3b pN0 L1	yes	no
116	56	06.08.2012	156	7	9.40	n/a	yes	pT3a pN1 cM0 L1 V0 Pn0	yes	no
117	77	07.08.2012	109	9	3.14	205	yes	pT3 pN1 (1/20) L1 V0 R0	yes	yes
118	66	09.08.2012	97	8	445.00	37	yes	pT2b pN0 cM0	n/a	yes
119	60	14.08.2012	105	7	57.90	n/a	yes	N1	yes	yes
120	77	16.08.2012	76	8	1000.00	n/a	yes	G2b	n/a	yes
121	73	20.08.2012	173	9	0.88	n/a	yes	T1c NX cM0	yes	yes
122	69	21.08.2012	97	7	13.90	490	no	T2b, cN0, cM0	yes	no
123	73	11.09.2012	119	8	16.20	183	no	T1c NX cM0	no	no
124	66	12.09.2012	87	6	0.55	252	no	n/a	n/a	no
125	68	17.09.2012	154	n/a	2.00	593	yes	n/a	no	no
126	72	21.09.2012	183	7	7.00	117	yes	pT3b pN0	yes	no
127	81	28.09.2012	120	7	7.39	970	no	T1c NX MX G2	no	no
128	63	02.10.2012	181	9	26.90	n/a	no	pT3a pN0 R1	yes	no
129	53	09.10.2012	207	9	0.15	n/a	yes	pT3, pN1	no	yes
130	71	10.10.2012	110	7	31.30	n/a	no	n/a	no	no
131	64	11.10.2012	141	7	0.19	n/a	yes	pT2c, pN0 (0/24)	no	no
132	69	12.10.2012	200	6	0.06	n/a	no	n/a	yes	yes
133	59	16.10.2012	152	7	7.60	219	no	n/a	yes	no
134	61	18.10.2012	148	8	8.55	n/a	yes	n/a	no	no
135	51	19.10.2012	159	7	0.67	138	yes	pT2c R0 N0 (0/19)	yes	no

136	72	22.10.2012	188	n/a	2.22	184	yes	pT2 pN0(0/7) cM0	no	no
137	76	23.10.2012	176	7	11.90	n/a	yes	pT2b pN0 L1 M0	yes	no
138	56	24.10.2012	95	n/a	1.50	n/a	yes	n/a	no	no
139	62	30.10.2012	235	7	0.46	118	yes	pT2b pN0 M0 R0	yes	no
140	76	08.11.2012	118	n/a	6.31	151	yes	n/a	yes	no
141	63	09.11.2012	222	n/a	8.16	785	no	n/a	no	no
142	71	14.11.2012	98	n/a	9.20	493	no	n/a	no	no
143	58	19.11.2012	185	9	6.24	10	yes	pT3a, pN0 G3 L0 R1	yes	no
144	63	20.11.2012	117	7	0.72	49	yes	pT2c pN1(0/12) R1	no	no
145	75	23.11.2012	190	9	9.38	10	no	T2 Nx M0 G2	yes	yes
146	75	28.11.2012	97	9	3.81	91	yes	pT3b pN1 M0 cM0	yes	yes
147	74	30.11.2012	188	7	7.86	85	yes	pT3b pN1(2/26) R1	no	yes
148	54	05.12.2012	121	7	2.45	183	yes	n/a	no	no
149	74	06.12.2012	148	7	3.77	74	yes	pT3a pN0 M0	yes	no
150	62	12.12.2012	140	8	12.10	n/a	no	n/a	yes	no
151	79	13.12.2012	67	6	15.00	47	yes	pT3a pN0	yes	no
152	63	17.12.2012	195	8	46.90	65	no	T3 N0 Mx	no	no
153	68	17.12.2012	260	n/a	13.60	469	no	n/a	no	no
154	75	03.01.2013	297	n/a	3.49	259	yes	pT2a N0 M0 R0 G2	no	no
155	65	08.01.2013	170	6	4.59	372	no	T2a N0 M09	yes	no
156	80	10.01.2013	123	7	5.55	n/a	yes	pT3b pN0 M0 G3	no	yes
157	71	14.01.2013	160	8	1.13	44	yes	pT3a pN0 pMx G3 R0	no	no
158	69	15.01.2013	237	8	2.39	14	yes	pT3b N1 (3/28) L1 R0	yes	no
159	77	15.01.2013	87	5	2.50	354	yes	pT2a R0 G2	yes	no
160	68	17.01.2013	157	7	14.10	n/a	yes	pT3b pN1 R0 G3a	yes	no
161	68	18.01.2013	84	9	68.70	105	yes	pT4 pN1 M0 R1 G3	yes	no
162	74	23.01.2013	166	7	4.24	69	yes	pT3b pN1 (1/19) M0 L1	n/a	yes
163	58	24.01.2013	76	6	0.51	70	yes	pT2b pN0 G2	yes	no
164	59	25.01.2013	104	n/a	63.30	n/a	no	n/a	yes	no
165	77	28.01.2013	239	8	11.60	206	nein	T2 N1 M0	yes	no
166	70	28.01.2013	133	7	2.56	46	nein	n/a	yes	no
167	77	29.01.2013	197	8	4.59	29	yes	pT2 pN0 M0	yes	no
168	76	31.01.2013	183	n/a	76.60	66	yes	n/a	no	yes
169	76	04.02.2013	241	9	1.13	154	yes	pT3b, ypN0 (0/19), R0	yes	no
170	63	05.02.2013	203	7	0.52	n/a	yes	pT3a pN0(0/3) Mx R1	yes	yes
171	78	06.02.2013	105	7	2.00	n/a	yes	pT3b pN0 Gl 7 R1	yes	yes
172	68	08.02.2013	171	7	18.50	17	no	T2a	no	no
173	63	11.02.2013	249	9	4.58	n/a	yes	pT3b pN1	yes	no
174	66	12.02.2013	142	9	3.98	n/a	no	n/a	n/a	yes
175	66	13.02.2013	122	n/a	3.75	n/a	no	n/a	yes	no
176	70	15.02.2013	186	7	2.77	86	yes	pT3a pN0 pMx V0 G3 R0	yes	no
177	70	19.02.2013	140	7	2.59	n/a	no	pT1c N0 M0	yes	no
178	81	20.02.2013	149	n/a	5.68	20	yes	pT3a pN0 M0 GII R1	yes	no
179	68	21.02.2013	169	7	1.65	954	no	n/a	yes	no
180	79	22.02.2013	126	8	1.19	n/a	yes	pT2 pN0 R0	yes	no
181	72	26.02.2013	125	8	2.60	39	yes	pT3a G3 pN0 pN1 R0	no	yes
182	64	27.02.2013	132	9	2.70	52	yes	pT3 pN0 Pn1 R1(0)	yes	no

183	76	28.02.2013	225	6	2.64	349	no	n/a	n/a	yes
184	66	01.03.2013	169	8	7.01	n/a	yes	pt3b cN0 V0 L1 Pn1 G3 R1	yes	no
185	72	05.03.2013	82	n/a	0.57	n/a	yes	pN0	no	no
186	65	06.03.2013	131	7	191.00	n/a	yes	pT3b R1	yes	yes
187	77	07.03.2013	205	6	7.00	169	yes	pT1b N1 M0	yes	no
188	64	08.03.2013	238	7	15.80	94	no	pT1c	yes	no
189	75	08.03.2013	217	8	2.62	20	yes	pT2b pN1(3/34) R1	yes	no
190	73	11.03.2013	222	9	104.00	47	no	n/a	no	no
191	67	13.03.2013	169	8	2.40	n/a	yes	pT2c N0 R1	yes	no
192	68	14.03.2013	187	9	7.20	2256	yes	pT3b pN1 G3 R0	yes	yes
193	74	15.03.2013	194	8	14.80	30	no	n/a	no	no
194	77	27.03.2013	120	7	29.70	n/a	yes	pT3a pN0 G3	yes	no
195	72	28.03.2013	174	n/a	21.40	n/a	yes	n/a	no	yes
196	68	03.04.2013	244	n/a	4.10	30	no	n/a	no	yes
197	60	03.04.2013	100	n/a	275.00	33	no	n/a	no	yes
198	69	04.04.2013	160	8	33.50	155	yes	pT3a, Nx, Mx, R1 G3a	yes	no
199	71	08.04.2013	166	7	20.70	n/a	no	n/a	no	no
200	78	08.04.2013	121	8	21.70	n/a	yes	pT2a pN0 M0	no	no
201	70	08.04.2013	201	9	0.86	56	yes	pT2 pN0 M0 R1	no	no
202	65	10.04.2013	107	7	2.13	19	yes	pT3b pN0 pM0 pV0 pL1	yes	no
203	53	10.04.2013	163	7	6.80	n/a	no	pT1c pN1 G3	no	no
204	62	12.04.2013	249	9	1.88	99	yes	n/a	no	no
205	66	15.04.2013	149	n/a	0.63	n/a	yes	pT3b pN1 R1	yes	no
206	61	18.04.2013	125	n/a	1.64	131	yes	pT3a pN0 G2 R1	yes	no
207	67	19.04.2013	115	n/a	47.40	n/a	no	n/a	n/a	no
208	63	22.04.2013	240	9	3.20	n/a	no	n/a	no	no
209	73	23.04.2013	73	7	6.43	91	no	n/a	n/a	no
210	65	26.04.2013	222	7	0.82	74	yes	n/a	yes	no
211	71	29.04.2013	84	6	2.61	1718	yes	pT2 Nx Mx	no	no
212	64	29.04.2013	111	n/a	8.01	205	no	n/a	yes	no
213	64	03.05.2013	274	7	1.35	238	yes	pT3a pN0	no	no
214	71	08.05.2013	153	6	61.20	n/a	no	n/a	no	no
215	72	15.05.2013	185	7	5.40	6	yes	pT3a pN0 M0 R0 G3	yes	no
216	77	16.05.2013	119	7	3.22	51	yes	pT2c pN0 M0 R0 G3	no	no
217	51	17.05.2013	240	7	0.78	n/a	yes	pT2c pN1(4/42) R1	yes	yes
218	62	22.05.2013	152	8	9.92	72	no	cT2c cN1 Mx St IV	yes	yes
219	62	23.05.2013	136	7	1.33	n/a	yes	pT3a G3 pN0 Pn1 R0	no	no
220	57	24.05.2013	195	7	4.49	241	no	pT2a T1c G2	yes	no
221	67	28.05.2013	134	8	0.55	74	yes	n/a	no	no
222	68	29.05.2013	129	9	36.00	109	no	n/a	no	no
223	70	04.06.2013	95	6	21.90	676	no	n/a	no	no
224	63	05.06.2013	102	8	6.03	17	yes	pT2c G3a	no	no
225	69	06.06.2013	142	7	5.45	n/a	yes	pT3a pN0 R0 cM0	yes	no
226	60	07.06.2013	120	7	267.00	53	no	n/a	no	no
227	56	12.06.2013	112	7	4.91	795	no	n/a	n/a	no
228	63	13.06.2013	212	7	3.18	14612	yes	n/a	yes	yes
229	72	13.06.2013	160	5	7.40	143	yes	pT2b pN0 M0	yes	no

230	61	14.06.2013	140	9	1.50	n/a	yes	n/a	yes	yes
231	66	19.06.2013	82	9	0.59	55	yes	pT3b pN1 (1/19) PN1	yes	no
232	71	19.06.2013	125	7	0.80	378	yes	pT2c pN0 R1 L0 V0	no	no
233	55	21.06.2013	180	7	25.93	33	no	pT3a pN1(4/17) R1 Pn1	no	no
234	66	24.06.2013	269	n/a	14.99	563	no	n/a	yes	no
235	65	24.06.2013	265	7	9.38	235	yes	n/a	yes	no
236	57	26.06.2013	178	7	8.00	n/a	no	n/a	no	yes
237	67	27.06.2013	104	7	0.39	16	yes	T2b, cN0, cM0	no	no
238	70	28.06.2013	174	7	14.90	n/a	no	T3b, pN0	no	yes
239	70	03.07.2013	66	8	1.05	29	yes	pT2c M0 N0 V0 R0	yes	no
240	71	09.07.2013	165	10	44.69	49	no	pT1c	yes	yes
241	60	10.07.2013	267	6	3.78	103	yes	pT3b pN1 R1 G3	yes	yes
242	63	11.07.2013	115	7	0.74	n/a	yes	pT2c pN0	no	no
243	77	12.07.2013	129	7	1.04	n/a	no	n/a	yes	yes
244	69	17.07.2013	117	7	n/a	n/a	yes	pT3b N0 pR1	yes	no
245	75	18.07.2013	66	8	0.51	n/a	yes	T2 N0 M0	yes	no
246	80	23.07.2013	97	9	0.14	12	no	n/a	no	no
247	64	27.07.2013	101	7	2.70	599	yes	pT3a N0 M0 R0	yes	yes
248	53	25.07.2013	178	9	1.90	123	yes	n/a	yes	no
249	65	26.07.2013	175	7	0.83	n/a	yes	pT3b pN1(2/13) M0 R0	yes	yes
250	58	30.07.2013	168	7	36.17	n/a	no	n/a	no	no
251	58	31.07.2013	87	9	0.38	n/a	yes	T3b, pN0 (0/33)	no	no
252	61	31.07.2013	147	9	1.35	309	yes	pT3a N0 (0/8) M0 R1	yes	no
253	76	01.08.2013	109	9	2.47	23	no	n/a	yes	no
254	61	05.08.2013	141	6	6.38	n/a	no	n/a	no	no
255	63	06.08.2013	313	8	0.27	849	yes	pT3b pN0 M0 R0	yes	no
256	65	06.08.2013	313	5	3.11	n/a	yes	pT3b pN1 R1 G3	no	no
257	72	07.08.2013	349	7	0.14	59	yes	pT3b pN1 L1 (1/8) V1 R1 G3	yes	yes
258	72	07.08.2013	261	7	2.38	205	yes	pT3a	yes	yes
259	71	08.08.2013	345	7	1.85	n/a	yes	n/a	yes	yes
260	60	08.08.2013	375	7	0.48	1221	yes	n/a	yes	no
261	63	12.08.2013	259	7	0.14	n/a	yes	pT2a pN0 cM0 R0	no	no
262	73	13.08.2013	261	8	16.00	n/a	no	n/a	yes	no
263	65	13.08.2013	304	9	5.21	30	no	pT4, pN1 (1/15), pn1, R1	yes	yes
264	57	13.08.2013	364	7	8.16	257	yes	pT3a pN1 cM0 Pn1 L1 R1	yes	no
265	71	14.08.2013	317	8	1.40	333	yes	pT2b pN0 R0 G3 a, pN1	yes	no
266	66	14.08.2013	400	7	0.10	50	yes	pT2c, pN0, R1	no	no
267	76	15.08.2013	314	9	2.35	131	yes	pT3b pN1 (3/7) L1 M0	yes	yes
268	75	15.08.2013	388	7	2.81	190	no	cT3b cN0 M0 G3	yes	no
269	74	16.08.2013	283	9	3.96	46	yes	n/a	no	no
270	77	19.08.2013	345	8	1.29	255	yes	pT2a N0	yes	no
271	72	20.08.2013	338	9	7.80	n/a	no	T2c cN0	no	no
272	73	23.08.2013	269	7	3.21	76	yes	pT2	yes	no
273	73	28.08.2013	241	7	6.00	n/a	yes	pT2b pN0 M0	no	no
274	68	28.08.2013	190	9	4.10	28	yes	pT4, pN1, L1, M0, R0	no	no
275	56	29.08.2013	253	7	2.39	445	yes	pT3b pN0	yes	no
276	73	04.09.2013	334	7	21.13	125	yes	pT3b, pN0, G3a	yes	yes

277	78	05.09.2013	336	5	4.08	1225	yes	pT2 N0 G2a	yes	no
278	54	10.09.2013	218	7	1.14	75	yes	pT2a pN0 M0 G3	yes	no
279	73	11.09.2013	236	n/a	13.84	n/a	no	n/a	no	no
280	75	12.09.2013	322	7	15.29	12	yes	n/a	no	no
281	77	16.09.2013	68	n/a	2.43	23	yes	n/a	no	no
282	57	17.09.2013	188	7	5.08	1399	no	n/a	no	no
283	81	18.09.2013	233	7	0.14	n/a	yes	pT3a, pN0, Mx, R0	no	no
284	55	20.09.2013	246	9	39.17	137	no	cT4, cN1, cM1b, G3	n/a	yes
285	67	24.09.2013	268	8	0.28	261	yes	pT2c pN0(0/11) M0 R0	no	no
286	67	24.09.2013	116	7	2.80	35	yes	pT2b, pN0, M0, GII	yes	no
287	53	25.09.2013	126	7	1.35	n/a	yes	pT3b N0 R1	no	yes
288	71	25.09.2013	305	8	2.70	n/a	yes	pT3a pN0	yes	yes
289	62	27.09.2013	292	7	24.73	373	yes	n/a	yes	no
290	72	27.09.2013	229	9	9.12	43	yes	pT3b pN1 cM0 G3 R1	no	yes
291	63	02.10.2013	240	6	0.62	122	yes	pT2c pN0 G2	yes	yes
292	66	04.10.2013	312	6	8.62	n/a	no	n/a	no	no
293	69	09.10.2013	223	9	1.01	45	yes	pT3a N0 cM0 L1 V0 R0 G3	no	no
294	62	14.10.2013	217	9	139.00	56	yes	pT4 pN1 (8/15) R1 L1	no	yes
295	74	22.10.2013	223	9	41395.00	10	yes	pT3b pN1	no	yes
296	58	28.10.2013	251	n/a	17.00	108	no	n/a	no	no
297	65	15.11.2013	156	7	0.81	n/a	yes	n/a	yes	no
298	72	18.11.2013	252	7	7.63	n/a	yes	pT2a G2b M0 Nx	yes	no
299	72	20.11.2013	275	9	113.81	206	yes	pT2a G3 pNx pMx	n/a	yes
300	73	22.11.2013	200	9	18.97	263	yes	n/a	no	no
301	78	25.11.2013	204	7	4.77	959	yes	n/a	no	no
302	74	25.11.2013	230	n/a	17.09	96	yes	pT3b pN1 (18/47) R1 L1 Pn1	no	no
303	76	26.11.2013	250	6	7.24	68	yes	n/a	n/a	no
304	79	26.11.2013	163	9	3.20	n/a	yes	pT3a pN0 cM0	yes	no
305	64	03.12.2013	220	7	10.76	621	yes	pT2a N0	no	no
306	46	03.09.2013	200	n/a	n/a	n/a	no	n/a	no	no
307	60	10.12.2013	336	7	1.82	n/a	yes	pT3b pN0 Mx	yes	no
308	72	11.12.2013	102	7	1.8	349	yes	pT2c pN1 M0 G3	yes	no
309	69	11.12.2012	200	n/a	4.39	292	no	n/a	no	no
310	68	12.12.2013	177	7	0.64	223	yes	n/a	no	no
311	72	13.12.2013	277	6	3.77	n/a	no	pT1c	yes	no
312	65	19.12.2013	170	7	1.49	1323	yes	pT3b	yes	no
313	71	17.12.2013	222	5	0.77	n/a	yes	pT2 pN0 cM0 G2	no	no
314	68	09.01.2014	163	n/a	12.9	n/a	yes	n/a	yes	no
315	57	09.01.2014	227	8	13.63	n/a	no	n/a	n/a	yes
316	76	10.01.2014	264	n/a	7.44	n/a	no	n/a	no	no
317	72	10.01.2014	194	n/a	313.13	n/a	n/a	n/a	n/a	no
318	72	15.01.2014	168	n/a	21.25	n/a	yes	n/a	no	no
319	75	28.01.2014	221	7	n/a	n/a	yes	pT2c N0 M0 R0 L0 V0	no	no
