# Supplementary figures and images for: The diagnostic value of PET/CT imaging with the 68Ga-labelled PSMA ligand HBED-CC in the diagnosis of recurrent prostate cancer
Source: Eur J Nucl Med Mol Imaging. 2014 Nov 20;42(2):197–209. doi: 10.1007/s00259-014-2949-6 (PMC4315487; doi:10.1007/s00259-014-2949-6)

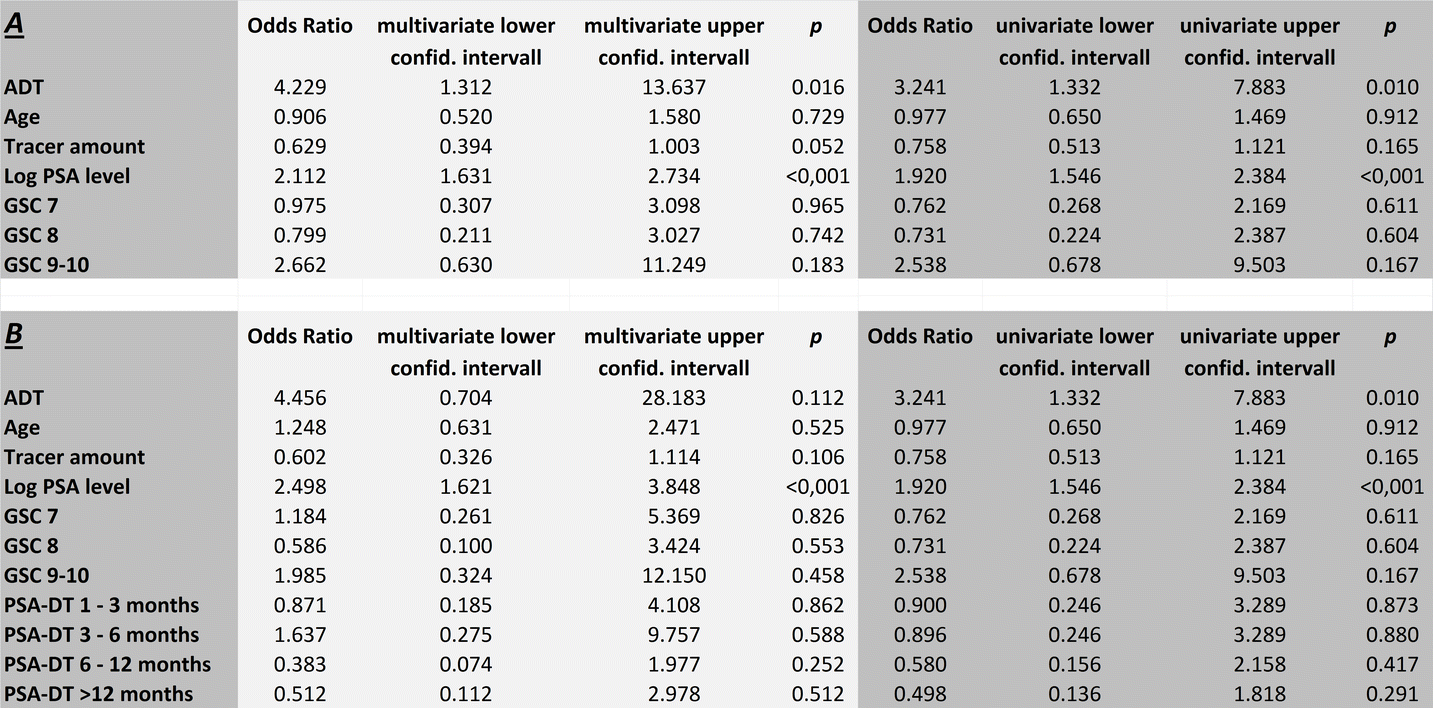

Supplement: Supplementary file 2 — Part A (upper table): results of the univariate and multivariate analyses for several variables possibly interacting with 68Ga-PSMA PET/CT. The number of patients for whom all listed variables are available is 277. Part B (lower table): same analyses as above but extended by several PSA doubling times (PSA DT). By including the PSA DT, the number of patients for whom all listed variables are available drops to 171. ADT androgen deprivation therapy, GSC Gleason score. (GIF 318 kb) [file 259_2014_2949_Fig6_ESM.gif]

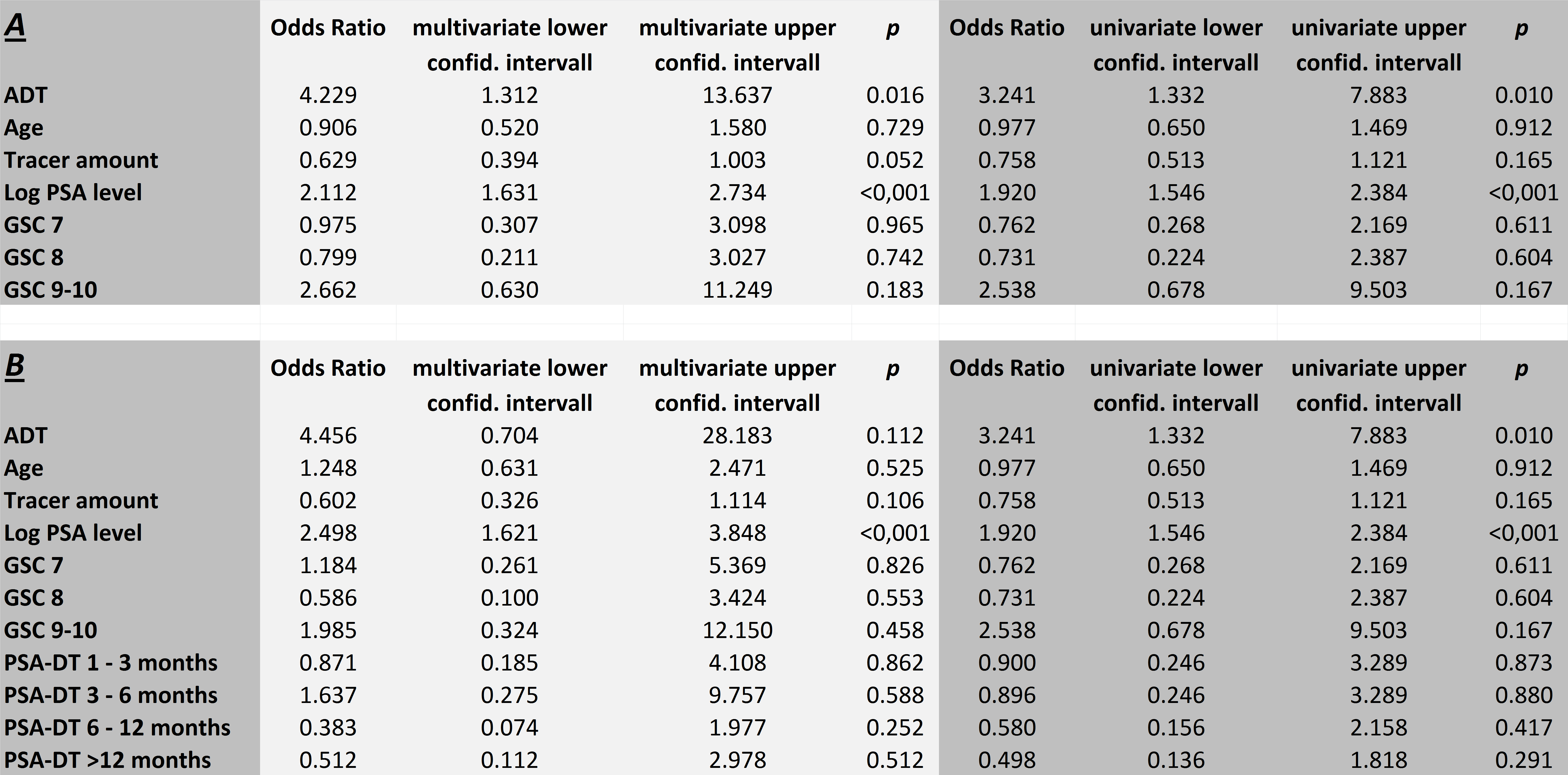

Supplement: Supplementary file 3 — High-resolution image (TIF 2.64 mb) [file 259_2014_2949_MOESM2_ESM.tif]

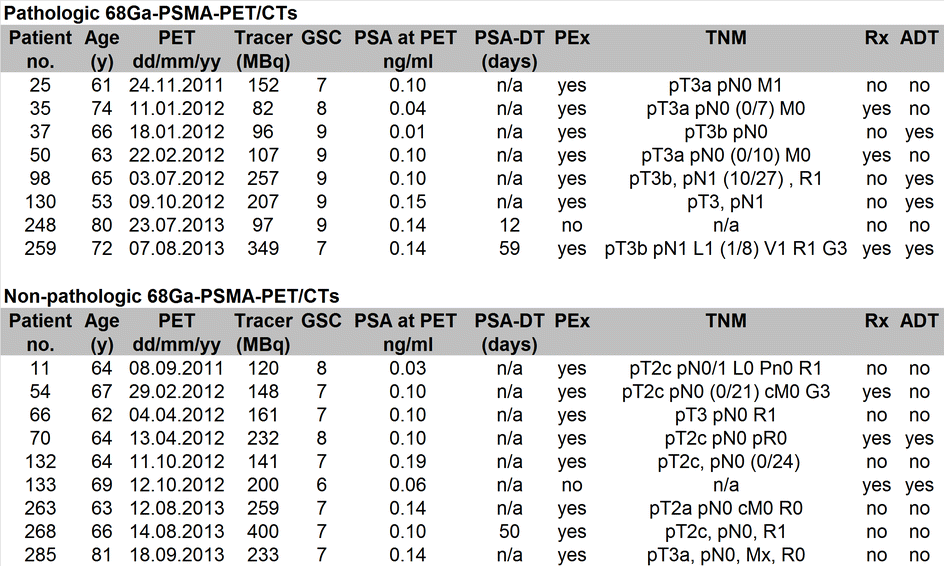

Supplement: Supplementary file 4 — Characteristics of patients with PSA values < 0.2 ng/ml. (GIF 123 kb) [file 259_2014_2949_Fig7_ESM.gif]

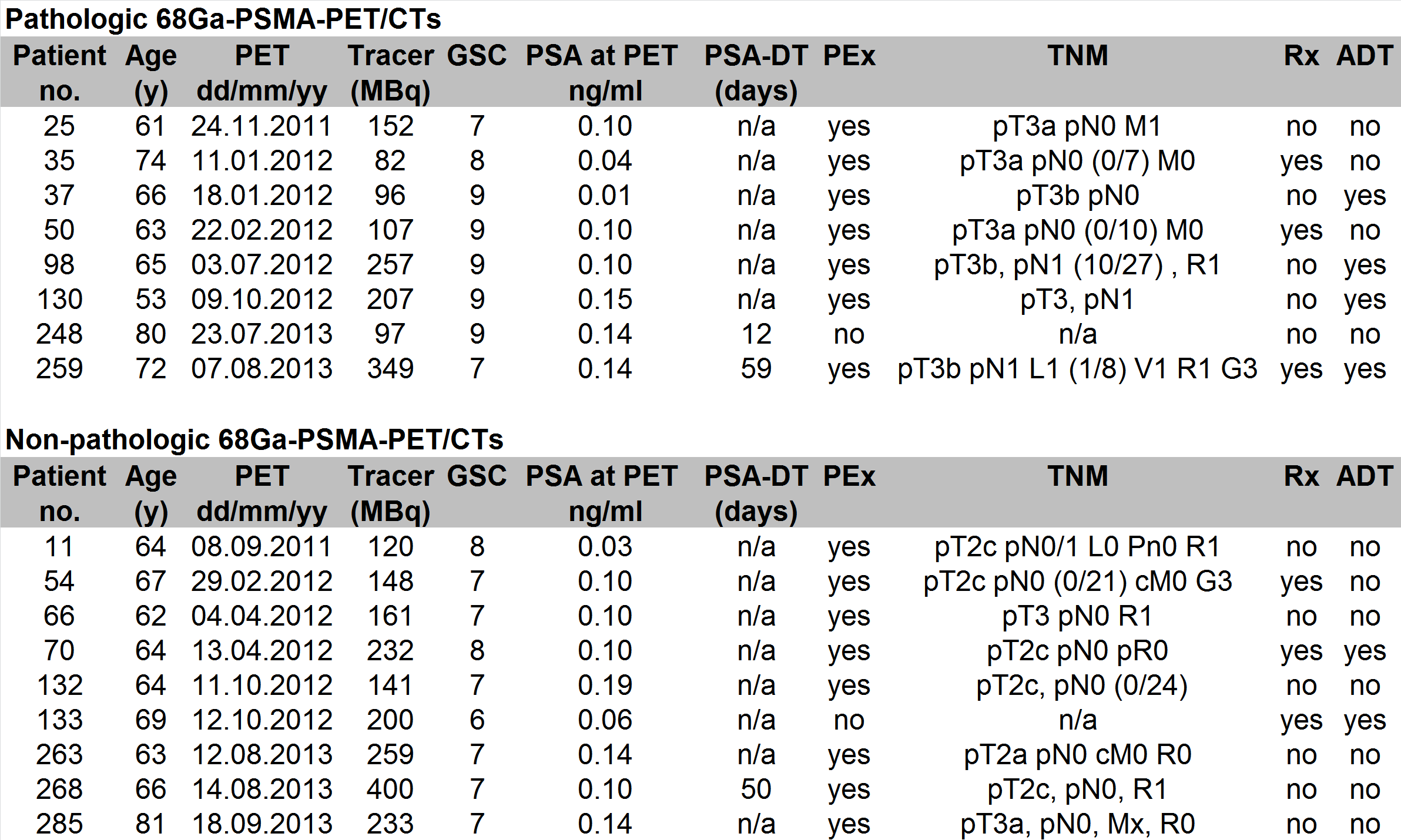

Supplement: Supplementary file 5 — High-resolution image (TIF 551 kb) [file 259_2014_2949_MOESM3_ESM.tif]
